# Global healthcare fairness: We should be sharing more, not less, data

**DOI:** 10.1371/journal.pdig.0000102

**Published:** 2022-10-06

**Authors:** Kenneth P. Seastedt, Patrick Schwab, Zach O’Brien, Edith Wakida, Karen Herrera, Portia Grace F. Marcelo, Louis Agha-Mir-Salim, Xavier Borrat Frigola, Emily Boardman Ndulue, Alvin Marcelo, Leo Anthony Celi

**Affiliations:** 1 Beth Israel Deaconess Medical Center, Department of Surgery, Harvard Medical School, Boston, Massachusetts, United States of America; 2 GlaxoSmithKline, Artificial Intelligence & Machine Learning, Zug, Switzerland; 3 Australian and New Zealand Intensive Care Research Centre (ANZIC-RC), Department of Epidemiology and Preventive Medicine, Monash University, Melbourne, Victoria, Australia; 4 Mbarara University of Science and Technology, Mbarara, Uganda; 5 Quality and Patient Safety, Hospital Militar, Managua, Nicaragua; 6 Department of Family & Community Medicine, University of the Philippines, Manila, Philippines; 7 Institute of Medical Informatics, Charité—Universitätsmedizin Berlin (corporate member of Freie Universität Berlin, Humboldt-Universität zu Berlin, and Berlin Institute of Health), Berlin, Germany; 8 Laboratory for Computational Physiology, Harvard-MIT Division of Health Sciences & Technology, Cambridge, Massachusetts, United States of America; 9 Anesthesiology and Critical Care Department, Hospital Clinic de Barcelona, Barcelona, Spain; 10 Department of Journalism, Northeastern University, Boston, Massachusetts, United States of America; 11 Department of Surgery, University of the Philippines, Manila, Philippines; 12 Department of Medicine, Beth Israel Deaconess Medical Center, Harvard Medical School, Boston, Massachusetts, United States of America; 13 Department of Biostatistics Harvard T.H, Chan School of Public Health, Boston, Massachusetts, United States of America; Yale School of Medicine: Yale University School of Medicine, UNITED STATES

## Abstract

The availability of large, deidentified health datasets has enabled significant innovation in using machine learning (ML) to better understand patients and their diseases. However, questions remain regarding the true privacy of this data, patient control over their data, and how we regulate data sharing in a way that that does not encumber progress or further potentiate biases for underrepresented populations. After reviewing the literature on potential reidentifications of patients in publicly available datasets, we argue that the cost—measured in terms of access to future medical innovations and clinical software—of slowing ML progress is too great to limit sharing data through large publicly available databases for concerns of imperfect data anonymization. This cost is especially great for developing countries where the barriers preventing inclusion in such databases will continue to rise, further excluding these populations and increasing existing biases that favor high-income countries. Preventing artificial intelligence’s progress towards precision medicine and sliding back to clinical practice dogma may pose a larger threat than concerns of *potential* patient reidentification within publicly available datasets. While the risk to patient privacy should be minimized, we believe this risk will never be zero, and society has to determine an acceptable risk threshold below which data sharing can occur—for the benefit of a global medical knowledge system.

## Introduction

Many widely available imaging datasets exist containing deidentified data from thousands of patients and may be used to train machine learning (ML) algorithms, such as the COVID-19 Chest X-ray Dataset Initiative [1] and the CheXpert Chest Radiograph dataset [2]. In the ideal case, open datasets provide a robust and diverse foundation to train clinical prediction models, leading to improved predictive accuracy and generalizability of the derived models. However, questions remain regarding the true privacy of publicly available deidentified health data, patient control over their data, and how we regulate data sharing in a way that that does not encumber progress or further potentiate biases for underrepresented populations throughout the world.

In this review article, we address concerns over data anonymization and further explore how increased regulations may inadvertently exclude developing countries over concerns of imperfect data anonymization. We argue limiting data sharing would not only slow the development of future medical innovations and clinical software, but could also potentially expand existing biases that favor high-income countries. While the risks to patient privacy should be minimized, we believe this risk will never be zero, and an acceptable risk threshold below which data sharing can occur must be agreed upon by society for the benefit of a global medical knowledge system.

## Deidentified health datasets promote innovation

The benefit of sharing deidentified data can be readily demonstrated by the widely used and publicly available Medical Information Mart for Intensive Care (MIMIC) database, now available in its fourth iteration [3,4]. This dataset includes deidentified clinical data from over 50,000 admissions to critical care units at Beth Israel Deaconess Medical Center in Boston, Massachusetts, United States of America, spanning over a decade in time. Access to the database requires a “Data Use Agreement,” which mandates that the developed source code for projects utilizing the data must be shared, promoting reproducibility and collaboration between research groups. Thousands of publications and conference proceedings have utilized this repository to advance our knowledge of critical care, and it has inspired the creation of similar databases in countries throughout the world, fostering international data sharing. The Society of Critical Care Medicine (SCCM) and the European Society of Intensive Care Medicine (ESICM) have embraced ICU patient data sharing [5], and the Amsterdam University Medical Center Database (AmsterdamUMCdb) adds to a growing list of globally available databases [6].

Further examples of how large international datasets have accelerated healthcare innovation continue to surface. One example includes a combination of mammography datasets from South Korea, USA, and the UK that led to the development of an AI algorithm that demonstrated not only improved breast cancer detection on mammography compared to radiologists, but also improved radiologist performance when assisted by AI [7]. Another example involves the use of open-access datasets and data from Stanford University—creating a dataset 2 orders of magnitude larger than previous skin pathology datasets—to develop a convolutional neural network (CNN) capable of skin cancer classification comparable to dermatology experts with potentially greater generalizability due to the size of the dataset enabling a more representative coverage of patients [8]. Similarly, combined datasets from China and the US were used to create algorithms capable of classifying macular degeneration and diabetic macular edema [9].

**Fig 1:**
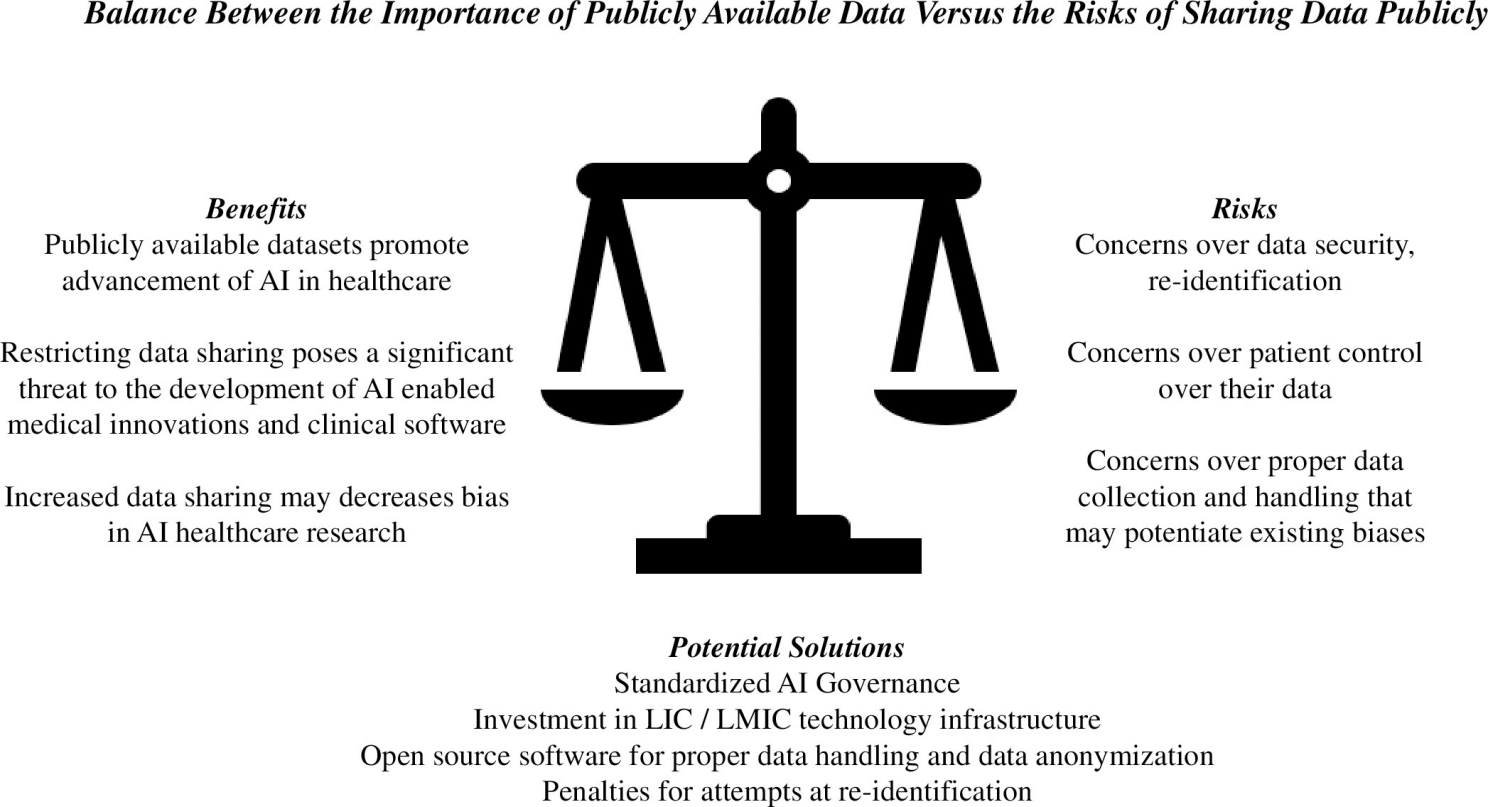
The balance between the importance of publicly available data versus the risks of sharing data publicly. Potential solutions are presented. AI, artificial intelligence; LIC, low-income country; LMIC, low-middle-income country.

Shared learning from these large datasets continue to provide powerful potential to enable scientific advances and medical innovation. However, many barriers exist to the creation of large, publicly available datasets, including concerns over the security of the shared data and how the open dataset will be used (Fig 1).

## Concerns over publicly available dataset security and regulation

In the ideal case, publicly available, deidentified datasets provide a robust and diverse foundation to train clinical prediction models, improving predictive accuracy and generalizability of the derived models and enabling medical innovation. However, concerns over the security and privacy of these datasets exist, potentially limiting the creation and diversity of new datasets. Interest in governmental regulation of these datasets is becoming more prevalent, as demonstrated by proposed laws in the US [10], China [11], and the European Union [12]. Patients are also becoming increasingly aware that their data are being used for research or commercial purposes and are rightfully more interested (particularly women and minorities) in controlling that data and how it is used [13]. One study found that most patients want to know what their personal health information (PHI) will be used for and are uncomfortable sharing their PHI with commercial entities but are comfortable sharing with their own institution [14]. Younger patients and women preferred more control of their PHI than older patients and males with respect to research participation, with a significant number of patients preferring study-specific consents. Most wanted to be informed of what their PHI was being used for, and 70% wanted to receive the results of studies using their PHI—highlighting the shifting patient preferences for more transparency and interest in how their PHI is being used.

Concerning data security, best practices [15] exist in the US for deidentifying image data to comply with the standards outlined in the Health Information Portability and Accountability Act (HIPAA) Privacy Rule [16], protecting a patient from potentially being identified from publicly available images. As defined in the HIPAA Privacy Rule, deidentified data are not regulated and may be shared without restriction. Standard deidentification techniques to remove protected information from images and associated metadata include pseudo-anonymization and k-anonymity [17,18]. However, deidentification methods are often automated and can be imperfect, as demonstrated, for example, by Google canceling the release of a public chest X-ray (CXR) dataset after discovering patient data was still embedded in some of the images [19].

Beyond the risk of leaking private information directly with an insufficiently deidentified dataset, there also exists the risk that an attacker may attempt to utilize information present in other publicly or privately available datasets to reestablish a link between deidentified patient images and their individual identities. One group has recently presented an ML approach that further highlights the linkage risk inherent in publicly available imaging datasets. Instead of training models on the available data to potentially predict pathology, they trained deep-learning (DL) models to identify patients from the available images. Their results are revealing: Multiple images of a single patient *can* accurately be determined to belong to the same original patient, despite standard deidentification efforts and without a shared identifier linking these images together [20]. Using 2 Siamese neural networks (SNN) trained on the Chest X-ray 14 dataset [21], the authors were able to determine whether 2 CXRs belonged to the same patient, even if taken several years apart and with new pathological development between the time points the images were taken. The implications are far-reaching: Given a patient’s CXR image, one could potentially match that image to other publicly or privately available CXR datasets that may contain imperfectly deidentified metadata and reidentify that patient or gain access to additional sensitive information. The ability to accurately match patients across deidentified datasets exposes the weakness of relying on deidentification techniques that do not guarantee complete anonymization or differential privacy [22]. Beyond reidentification from CXR images, additional concerns exist regarding reidentification from head and neck images that include the patient’s face, highlighting the need for defacing software to deface patient images in datasets that include facial profiles [23,24].

An important consideration is the trade-off between the degree of anonymization—as measured in terms of differential privacy—and utility/representativeness for downstream clinical prediction tasks [25]. Despite Packhauser and colleagues demonstrating a potential route for an attacker to gain access to sensitive patient information, it is essential to note that the mere ability to match records belonging to the same patient does not yet constitute a reidentification. An attacker would still require access to either (i) an imperfectly deidentified dataset that allows further inferences about the patient or their identity or (ii) a dataset that was not deidentified containing private information about the patient to be able to exploit the ability to match patients across datasets [26].

## Tempering concerns over public data security

Despite these valid concerns over data security, there currently exists little publicly available evidence of patient identities having been linked to open health data (OHD) despite the theoretical impossibility of true anonymization. “Nothing about an individual should be learnable from the database that cannot be learned without access to the database” was, to the best of our knowledge, first proven by Dwork [27] and has led to the development of the differential privacy framework that—in lieu of full anonymization—instead seeks to quantify and provide theoretical limits for the maximum privacy loss incurred by individuals. Dwork’s impossibility result has far reaching consequences for healthcare practitioners wishing to release *any* data. In particular, reidentification attacks, such as the one highlighted in [20], cannot be ruled out fully due to auxiliary information, and practitioners wishing to release healthcare data are therefore left with the difficult decision of managing the trade-off between the value to society of sharing data on the one hand and the risk of privacy loss for individuals on the other. While the previously mentioned HIPAA Privacy Rule outlines clear criteria for deidentification, it does not provide regulatory guidelines on managing differential privacy trade-offs. It is important to note that the potential for privacy loss and reidentification applies to the release of any data, including statistics on a cohort level. In response to these challenges, several organizations that routinely deal with personal data and statistics thereof have turned towards the adoption of differential privacy methods to systematically quantify and manage privacy risks, such as, for example, the US Census Bureau [28] and Apple [29].

To better quantify the risk of patient reidentification, we sought to evaluate the literature on this topic to assess current concerns over data security, including larger-scale data breaches. Subtle approaches to reidentification of (potentially improperly) anonymized health data stand in stark contrast to the illegal, forcible acquisition of personal health data by means of a data breach—which includes illegal disclosure, attainment, or use of information without authorization. Theft of medical records—in contrast to credit card records—is attractive to criminals because they contain sufficient information to secure loans, open a bank account, obtain health services and prescription medication, etc. In brief, identity theft may be the principal reason for intentional data disclosure [30]. Of reported data breaches in the USA between 2015 and 2019, the health sector accounted for 76% of all 10 billion cases [31].

**Fig 2:**
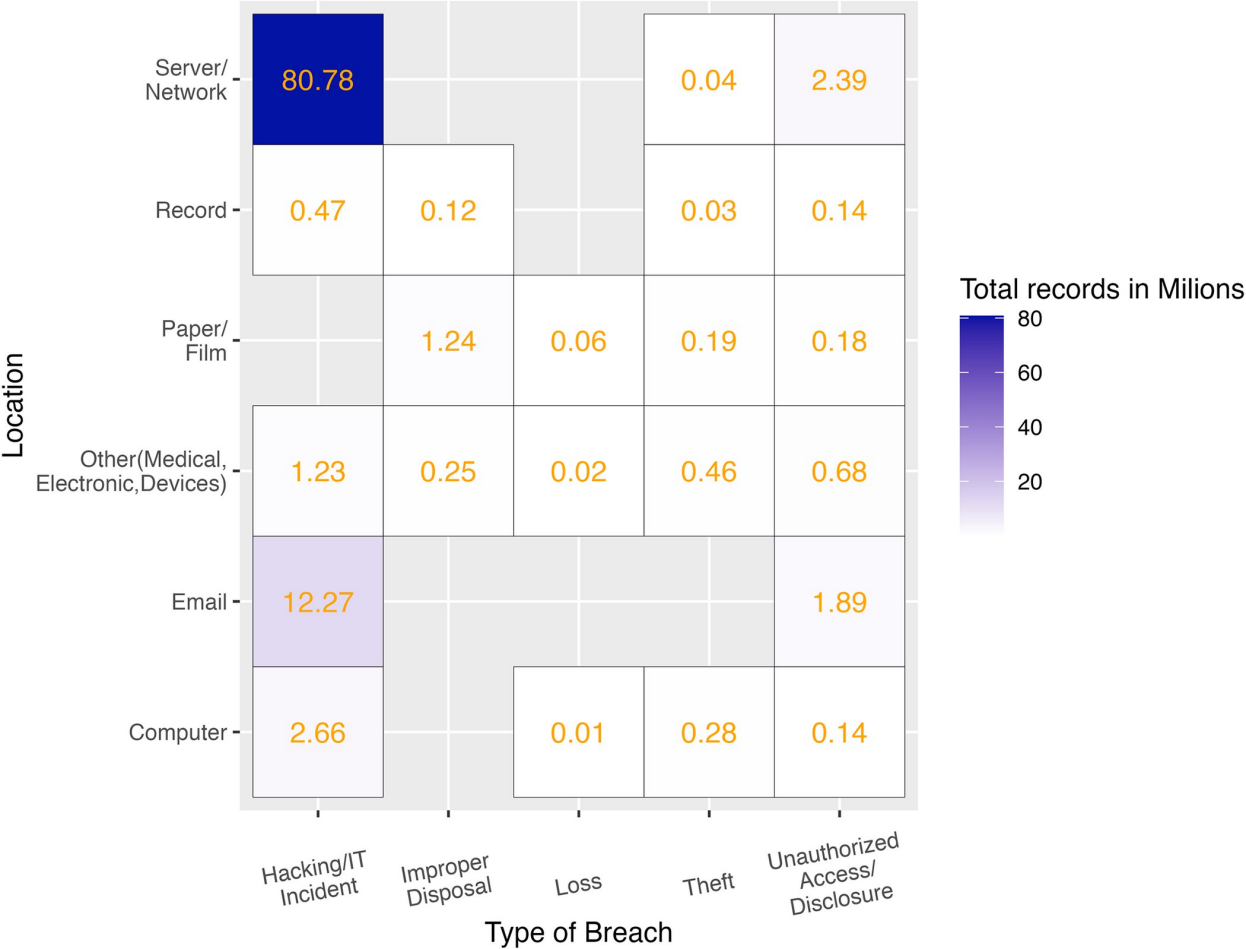
Magnitude of data breaches considering total number of records affected by type of breach and location in the US between November 2019 and November 2021. Elaborated from raw data [32].

Under US federal legislation, if a healthcare data breach affects 500 or more patients, it must be reported to the Office of Civil Rights (OCR). The OCR data breach portal provides an online database describing data breaches of protected health information (PHI) [32]. To evaluate the frequency of PHI data leakage through data breaches, we downloaded and analyzed data containing type of breach, location of breached information, and number of individuals affected during the last 2 years. The most frequent type of PHI data breach was the one produced by hacking activities (72%) followed by unauthorized disclosure (21%). Regarding where the data was hosted when the breach occurred, almost all leaks affected servers (93%) and the second location was email (35%). Network servers and email have become the main locations for hackers using different techniques such as malware, ransomware, or phishing attacks to prey on electronic health records (EHRs) [31]. Note that 62% of the data leaks came from more than 1 location. Combining type of breach and location, hacking the internal network to reach servers is the main cause of PHI data leakage well above other causes (Fig 2).

Compared to the large and growing problem of hackers attacking PHI data servers, there is no category nor series of case reports about data leakage from OHD. A potential reason for this discrepancy could be that anonymized OHD would generally be stripped of patients’ full names, address histories, financial information, and social security numbers, making OHD less valuable for criminal purposes than private datasets that include personalized information. Solely clinical data and “quasi-identifiers” remain that could potentially be reidentified with relatively greater effort when combined with other data sources that contain personal identifiers. In the end, a threat by criminal activity is present wherever data are stored and are always subject to misuse or theft—with the utility of the data for malicious uses being diminished the harder establishing a link to personalized identifiers is.

In order to approximate the number of known cases where individuals were reidentified from OHD, we performed a literature review on PubMed [query = (“case” OR “example”) AND (“re-identification” OR “reidentification” OR “re-id”) NOT (“theoretical” OR “video” OR “camera” OR “pedestrian” OR “visual”)], excluding studies on reidentification attempts from security cameras and genomic information (S1 Table). Reviewing the literature (*n* = 65) and other relevant articles, reports on individuals being publicly reidentified from OHD were scarce. A systematic review of reidentification attacks concluded the success rate of health data reidentification attempts was very low when considering studies that included data deidentified according to modern standards [33]. A contemporary analysis that assessed the reidentification risk from a healthcare dataset of over 1 million patients at Vanderbilt University Medical Center also suggests that reidentification risk is low considering a potential attacker’s resources and capabilities [34].

In order to get a more holistic view outside of the medical literature, we expanded our search by reviewing news coverage using Media Cloud, an open-source global news database and analysis tool [35]. Media Cloud covers news stories, blog entries, and web pages and regularly ingests news content from more than 60,000 publications worldwide. It also provides retrospective coverage for many sources going back to 2010. Our search was limited to the US only, as the use of this system requires careful selection of a media corpus to study. The well-bounded media “collection” of the US provides the ability to draw meaningful conclusions about the volume and proportion of attention to a specific topic within a larger media coverage “universe” instead of as a tool to simply find relevant articles. Including other global areas would have potentially diluted our ability to find meaningful results using this method, and the US media ecosystem is also known to be sensitive for media attention to subjects globally. For this survey, we searched over 10,000 media publications from the US, publishing at either the state or national level. The query used to identify coverage on this issue was [“de-anonymization” OR deanonymization OR “de-anonymize” OR deanonymize OR “re-identification” OR reidentification OR “re-identify” OR reidentify]. The timeframe of coverage searched was 5 years, 09-01-2016 through 09-01-2021. Data were manually cleaned to remove a minimal number of irrelevant articles (in which the term deanonymize was used in other contexts), or coverage to the issue outside of the US. The resulting corpus was 186 stories from 127 sources (S2 Table). We manually coded the articles on 2 variables: The first was the context in which the issue was discussed: theoretical discussion (143/186, 77%), discussion of research released (20/186, 11%), or discussion of an actual case (23/186, 12%). The remaining articles without cases or research were assigned the code of discussing the issue theoretically (i.e., in the abstract). Despite ongoing thematic coverage of this issue over the past 5 years, there is a paucity of actual examples (S3 Table). Hence, we can assume that if there were more publicly known examples, there would be considerable coverage given the ongoing news attention to this issue. None of the identified cases involved health data nor healthcare databases.

Against this backdrop, the medical field must address essential questions, such as how do we improve PHI protection, how do we increase patient involvement in how their data are used, and how do we do this in a way that continues to promote global collaborative efforts in analyzing OHD without a complete shutdown in progress? Given the paucity of evidence supporting concerns over data security of publicly available datasets, we believe that continued investment in publicly available datasets to promote innovation is prudent and that there may be implicit harm in limiting data sharing.

## Potential harms of prematurely limiting data sharing—Potentiating bias

The aforementioned developments suggest that matching deidentified patient records across datasets is potentially easier than previously thought, and patients are at the same time increasingly interested in more control over how their data are being used. Increased availability of deidentified patient data has led to a global boom [36] in innovation with ML-driven software as a medical device (SaMD), with little data to support concerns over publicly available medical data security. Crucial to this discussion is how limiting data sharing (such as the current legal framework proposed by the EU [12]) would affect underrepresented populations and potentiate bias [37].

The medical knowledge system that informs clinical practice worldwide has historically been based on studies primarily performed on a handful of high-income countries and typically enrolling white males [38–40]. Guidelines for the management of heart disease, for example, are disseminated to the rest of the world from professional societies such as the American Heart Association. To truly move towards a global knowledge medical system that incorporates data from all parts of the world to decrease bias and increase data fairness, data from places that historically have not had a leading role in the development of current medical standards should be included—ethnic minorities, lower-income countries (LICs), and lower-middle-income countries (LMICs). One example in which bias affected an ML algorithm includes a breast cancer histology algorithm that reflected ethnicity rather than intrinsic tumor biology due to site-specific staining protocols and region-specific demographics [41]. In this example, the bias was introduced due to 1 site having more black patients included than many of the other sites. Biased models risk repeating cancer care inequities related to ethnic background. Another study identified extensive bias in several publicly available CXR datasets used for ML algorithms and found multisource datasets may combat such bias [42]. There is evidence of bias and underrepresentation even within the US as 70% of data comes from 3 wealthy states as opposed to rural regions, and less than one-third of states are represented in data-sharing platforms [43].

With digitalization, every country has an opportunity to create its own medical knowledge system using data routinely collected in the process of care. However, many countries are only just starting to reach the levels of digitalization of countries like the US and China, and increased regulations on the use of AI could further limit the participation of developing countries in these global datasets. Up to 94% of funding for AI startups over the last 5 years is accounted for by the US and China [44]. This poses the risk of potentiating bias given the limited diversity of datasets. However, there could potentially be harm in using data from developing countries as well given concerns for poor data collection methods and lack of inclusion of disadvantaged populations. Indeed, biased AI has led to racial profiling in South Africa [45] and labor exploitation in Venezuela [46]. In Africa and Latin America, there is a significant lack of knowledge and regulatory frameworks surrounding PHI, and the concept of PHI is largely alien to the patient and sometimes practitioners [47,48]. Similarly, in the Philippines, illness and care is a communal experience, with many taking comfort in sharing the steps of their care process with family and close friends—which, in many rural areas without digital healthcare, is essentially the entire village or *barangay*. As many as 2.3 million Filipino families have no electricity, limiting access to digital healthcare. Sub-Saharan Africa (including Uganda) suffers from limited usage of electronic health records due to the high cost of procurement and maintenance, poor internet connectivity, intermittent power supply to the rural settings, and low uptake by healthcare workers [49,50]. There is limited training on how to protect and/or handle patient information as this is prioritized secondary to care delivery given technical challenges (e.g., power supply, internet, and computer infrastructure) and the requirement that a strained workforce must serve high volumes of patients [51].

The digital healthcare experience in these countries emphasizes how increased regulations on publicly available datasets will likely raise the barrier to entry for developing countries, further excluding their populations from datasets and increasing biases that favor high-income countries. While no data directly supports this at this time, there is some evidence that limiting data flow adversely affects innovation [52]. It is possible that barriers to data flow make it more time-intensive and expensive to share data overseas, benefiting those countries with the resources to overcome these barriers.

## Potential solutions

Therefore, if we are to achieve unbiased datasets that represent the global community, the leaders in healthcare digitization need to assist LICs/LMICs with contributing to publicly available datasets, but also assist in enabling accurate data collection. This support would allow these countries to leverage their data to solve clinical problems unique to their populations and improve current global datasets by more representatively covering diverse populations. As LICs and LMICs embark on developing digital health infrastructure, views of the marginalized and vulnerable must be included in defining how their data will be collected, used, and how they can benefit. It is therefore essential to engage patients and the community when promoting digital literacy in LICs/LMICs. Varied demographics—for example, laborers, the urban poor, indigenous peoples, elderly, women—must be involved in developing PHI governance bodies so that their voices are included.

Other proposed solutions to improving data sharing include promising new technologies, such as synthetic data or federated learning [53–55], which have been suggested to potentially help researchers publicly sharing health data while better managing the risk of deidentification. However, linkage risk will always remain a concern as even releasing summary statistics alone constitutes a certain loss of privacy for the contributing data sources in terms of differential privacy [28,56]. In the federated learning framework, investigators from different institutions combine efforts by training a model locally on their own data, and sharing the trained model parameters with others to generate a central model, rather than sharing the source data directly [57]. However, the feasibility of using federated learning for data sharing is predicated on consistent data curation, standards, and harmonization across the participating institutions. Additionally, given that the data are not combined, the opportunity to expand the number of rare events may not be fully leveraged if the modeling is performed in isolation and only the meta-model is shared across institutions. Most importantly, algorithmic bias will be harder to detect if local investigators only see their own data. Given how challenging it is to detect and fix algorithmic bias in models trained on pooled data, it would likely be even more difficult to address algorithmic bias when learning is distributed. Resource allocation towards federating learning platforms and technologies should therefore be balanced with those allocated towards better tools for deidentification and standardized data curation.

To ensure proper data collection and sharing, legal policy and data security frameworks should be put in place to strengthen the protection of PHI datasets from accidental leakage and potential malicious outside attacks [58]. These policies should in particular regulate stewards of PHI datasets that, if combined, may enable reidentification via linkage with openly available datasets. Substantial penalties should be developed to punish any attempts to exploit linkage of open medical data with the aim of reidentifying patients or using PHI for commercial purposes, rather than for society’s benefit, without patient consent. Additionally, although increasing patient involvement undoubtedly adds more complexity, patient stewardship over their data is a fundamental right. As the technology to study PHI advances, technologies to improve PHI management ought to advance in lockstep. Numerous countries have embarked on creating AI governance frameworks, but there is no central coordination between nations to set standards for proper handling of data sharing across international boundaries [59]. Equitable AI governance and attempts at global AI regulations and standards may help consider the needs and inequalities of developing countries [60].

Investment in technology infrastructure (such as EHRs) for data collection and data sharing and surveillance for this technology should be a priority for these populations. The benefits of global investment in LIC/LMIC digitalization are numerous and include improved accuracy of collected data through health information management systems, decreased bias, and improved algorithmic fairness through the inclusion of marginalized groups in training data and ensure accountability for proper data collection and sharing. To foster investment, developing countries may want to consider incentives for foreign tech companies to conduct research and develop facilities to promote infrastructure development. The global AI community should continue to consider these investments, creating open-source software to promote proper data handling, data anonymization software, and AI governance standards. Although MIMIC represents a model for the use of big data and how it may contribute to improving medical understanding in high-income countries, such a model may be less feasible in LICs/LMICs where patients may lack access to advanced clinical services. Thus, as health systems in LIC/LMICs undergo digital transformation, there should be equal attention to, or even affirmative action towards, data analysis of services rendered at the primary care level where the majority of clinical encounters for health promotion and disease prevention occur. In many cases, poor or socially disadvantaged patients may have more complex diseases and have no recourse but to receive care in the nearest public primary care facility, potentially never reaching a hospital. These patients would not be represented if data collection was limited to hospitalized patients, and, therefore, the structure of local healthcare systems must be considered when designing open clinical databases for LICs/LMICs.

## Conclusions

We would argue that the cost—measured in terms of access to future medical innovations and clinical software while potentiating bias—of slowing ML progress is too great to stop sharing data through large publicly available databases for concerns over imperfect anonymization and potential linkage risks. Although the potential for linking public medical records at the detriment of patients exists, a robust regulatory framework that protects both open sharing of deidentified data for good, and strongly penalizes patient reidentification, may be a more measured solution than attempting to broadly limit data sharing. Publicly available datasets provide the fuel for widespread application and adoption of AI in healthcare and for advancing our understanding of heterogeneous and diverse patient populations globally. Slowing progress by limiting data sharing risks curtailing medical innovation and significantly impeding our ability to advance our understanding of health and global disease. Preventing AI’s progress towards precision medicine and sliding back to the “white-size-fits-all” clinical practice dogma poses a more significant threat than contemporary concerns of *potential* patient reidentification within publicly available datasets. This potential reidentification risk will never be zero, and we have to determine an acceptable risk threshold for sharing data for the benefit of a more global medical knowledge system. The global AI community needs to take an active role to assist developing nations on their healthcare digitization quest through standardized AI governance for data sharing and investment in equitable data collection infrastructure.

## Supporting information

S1 Table Open health data reidentification PubMed review results. (PDF)

S2 Table Coded deanonymization and reidentification stories. (PDF)

S3 Table Complete list of individual cases on media about personal information disclosure. (PDF)
