# Supplementary material for: Global healthcare fairness: We should be sharing more, not less, data
Source: PLOS Digit Health. 2022 Oct 6;1(10):e0000102. doi: 10.1371/journal.pdig.0000102 (PMC9931202; doi:10.1371/journal.pdig.0000102)
Supplement: S1 Table — (PDF) [file pdig.0000102.s001.pdf]

Supplementary Table 1: Open Health Data Re-Identification PubMed Review Results

PMID	Title	Authors	Citation	First Author	Journal/Book	Publication Year	Create Date	PMCID	NIHMS ID	DOI
29991478	Genomic Privacy	Schwab AP, Luu HS, Wang J, Park JY.	Clin Chem. 2018 Dec;64(12):1696-1703. doi: 10.1373/clinchem.2018.289512. Epub 2018 Jul 10.	Schwab AP	Clin Chem	2018	2018/07/12			10.1373/clinchem.2018.289512
30629502	Progressive Learning for Person Re-Identification with One Example	Wu Y, Lin Y, Dong X, Yan Y, Bian W, Yang Y.	IEEE Trans Image Process. 2019 Jan 10. doi: 10.1109/TIP.2019.2891895. Online ahead of print.	Wu Y	IEEE Trans Image Process	2019	2019/01/11			10.1109/TIP.2019.2891895
30972355	Assessing and Minimizing Re-identification Risk in Research Data Derived from Health Care Records	Simon GE, Shortreed SM, Coley RY, Penfold RB, Rossom RC, Waitzfelder BE, Sanchez K, Lynch FL.	EGEMS (Wash DC). 2019 Mar 29;7(1):6. doi: 10.5334/egems.270.	Simon GE	EGEMS (Wash DC)	2019	2019/04/12	PMC6450246		10.5334/egems.270
33448306	Enabling realistic health data re-identification risk assessment through adversarial modeling	Xia W, Liu Y, Wan Z, Vorobeychik Y, Kantacioglu M, Nyemba S, Clayton EW, Malin BA.	J Am Med Inform Assoc. 2021 Mar 18;28(4):744-752. doi: 10.1093/jamia/ocaa327.	Xia W	J Am Med Inform Assoc	2021	2021/01/15			10.1093/jamia/ocaa327
32165110	[The risk of re-identification when analyzing electronic health records: a critical appraisal and possible solu	Hauswaldt J, Demmer I, Heinemann S, Himmel W, Hummers E, Pung J, Schlegelmilch F, Drepper J.	Z Evid Fortbild Qual Gesundhwes. 2019 Dec;149:22-31. doi: 10.1016/j.zefq.2020.01.002. Epub 2020 Mar 10.	Hauswaldt J	Z Evid Fortbild Qual Gesundhwes	2019	2020/03/14			10.1016/j.zefq.2020.01.002
33754988	Epidemiologic and Genomic Reidentification of Yaws, Liberia	Timothy JWS, Beale MA, Rogers E, Zaizay Z, Halliday KE, Mulbah T, Giddings RK, Walker SL, Thomson NR, Kollie KK, Pullan RL, Marks M.	Emerg Infect Dis. 2021 Apr;27(4):1123-1132. doi: 10.3201/eid2704.204442.	Timothy JWS	Emerg Infect Dis	2021	2021/03/23	PMC8007311		10.3201/eid2704.204442
33381836	The effect of kinship in re-identification attacks against genomic data sharing beacons	Ayoz K, Aysen M, Ayday E, Cicek AE.	Bioinformatics. 2020 Dec 30;36(Suppl_2):i903-i910. doi: 10.1093/bioinformatics/btaa821.	Ayoz K	Bioinformatics	2020	2020/12/31	PMC7773481		10.1093/bioinformatics/btaa821
30052749	Re-identification of individuals in genomic data-sharing beacons via allele inference	von Thenen N, Ayday E, Cicek AE.	Bioinformatics. 2019 Feb 1;35(3):365-371. doi: 10.1093/bioinformatics/bty643.	von Thenen N	Bioinformatics	2019	2018/07/28			10.1093/bioinformatics/bty643
31690260	European Medicines Agency Policy 0070: an exploratory review of data utility in clinical study reports for a	Ferran JM, Nevitt SJ.	BMC Med Res Methodol. 2019 Nov 5;19(1):204. doi: 10.1186/s12874-019-0836-3.	Ferran JM	BMC Med Res Methodol	2019	2019/11/07	PMC6833240		10.1186/s12874-019-0836-3
34077454	Fundamental privacy rights in a pandemic state	Carvalho T, Faria P, Antunes L, Moniz N.	PLoS One. 2021 Jun 2;16(6):e0252169. doi: 10.1371/journal.pone.0252169. eCollection 2021.	Carvalho T	PLoS One	2021	2021/06/02	PMC8171945		10.1371/journal.pone.0252169
33178000	Unsupervised Few-Shot Feature Learning via Self-Supervised Training	Ji Z, Zou X, Huang T, Wu S.	Front Comput Neurosci. 2020 Oct 14;14:83. doi: 10.3389/fncom.2020.00083. eCollection 2020.	Ji Z	Front Comput Neurosci	2020	2020/11/12	PMC7592391		10.3389/fncom.2020.00083
31922426	Privacy Risks of Sharing Data from Environmental Health Studies	Boronow KE, Perovich LJ, Sweeney L, Yoo JS, Rudel RA, Brown P, Brody JG.	Environ Health Perspect. 2020 Jan;128(1):17008. doi: 10.1289/EHP4817. Epub 2020 Jan 10.	Boronow KE	Environ Health Perspect	2020	2020/01/11	PMC7015543		10.1289/EHP4817
30106660	New and Improved? 21(st) Century Cures Act Revisions to Certificates of Confidentiality	Wolf LE, Beskow LM.	Am J Law Med. 2018 May;44(2-3):343-358. doi: 10.1177/0098858818789431.	Wolf LE	Am J Law Med	2018	2018/08/15	PMC6173954	NIHMS989590	10.1177/0098858818789431
30419918	Federating patients identities: the case of rare diseases	Maaroufi M, Landais P, Messiaen C, Jaulent MC, Choquet R.	Orphanet J Rare Dis. 2018 Nov 12;13(1):199. doi: 10.1186/s13023-018-0948-6.	Maaroufi M	Orphanet J Rare Dis	2018	2018/11/14	PMC6233538		10.1186/s13023-018-0948-6
31484555	Pseudonymization for research data collection: is the juice worth the squeeze?	Kohlmayer F, Lautenschläger R, Prasser F.	BMC Med Inform Decis Mak. 2019 Sep 4;19(1):178. doi: 10.1186/s12911-019-0905-x.	Kohlmayer F	BMC Med Inform Decis Mak	2019	2019/09/06	PMC6727563		10.1186/s12911-019-0905-x
34295512	Re-identification of individuals from images using spot constellations: a case study in Arctic charr (Salvelir	De Bicki IT, Mittell EA, Kristjánsson BK, Leblanc CA, Morrissey MB, Terzić K.	R Soc Open Sci. 2021 Jul 21;8(7):201768. doi: 10.1098/rsos.201768. eCollection 2021 Jul.	De Bicki IT	R Soc Open Sci	2021	2021/07/23	PMC8292754		10.1098/rsos.201768
34745953	Re-Identification of Patient Subgroups in Uveal Melanoma	Nguyen THY, Nguyen T, Nguyen QH, Le DH.	Front Oncol. 2021 Oct 20;11:731548. doi: 10.3389/fonc.2021.731548. eCollection 2021.	Nguyen THY	Front Oncol	2021	2021/11/08	PMC8564479		10.3389/fonc.2021.731548
32597785	Toward the Development of Data Governance Standards for Using Clinical Free-Text Data in Health Resea	Jones KH, Ford EM, Lea N, Griffiths LJ, Hassan L, Heys S, Squires E, Nenadic G.	J Med Internet Res. 2020 Jun 29;22(6):e16760. doi: 10.2196/16760.	Jones KH	J Med Internet Res	2020	2020/06/30	PMC7367542		10.2196/16760
22164229	A systematic review of re-identification attacks on health data	El Emam K, Jonker E, Arbuckle L, Malin B.	PLoS One. 2011;6(12):e28071. doi: 10.1371/journal.pone.0028071. Epub 2011 Dec 2.	El Emam K	PLoS One	2011	2011/12/14	PMC3229505		10.1371/journal.pone.0028071
32162281	Less than five is less than ideal: replacing the "less than 5 cell size" rule with a risk-based data disclosure	Wilkinson K, Green C, Nowicki D, Von Schindler C.	Can J Public Health. 2020 Oct;111(5):761-765. doi: 10.17269/s41997-020-00303-8. Epub 2020 Mar 11.	Wilkinson K	Can J Public Health	2020	2020/03/13	PMC7501321		10.17269/s41997-020-00303-8
27130179	Efficient and effective pruning strategies for health data de-identification	Prasser F, Kohlmayer F, Kuhn KA.	BMC Med Inform Decis Mak. 2016 Apr 30;16:49. doi: 10.1186/s12911-016-0287-2.	Prasser F	BMC Med Inform Decis Mak	2016	2016/05/01	PMC4851781		10.1186/s12911-016-0287-2
26262118	Patient-Centered Outcomes Research in Practice: The CAPriCORN Infrastructure	Solomonides A, Goel S, Hynes D, Silverstein JC, Hota B, Trick W, Angulo F, Price R, Sadhu E, Zelisko S, Fischer J, Furner B, Hamilton A, Phua	Stud Health Technol Inform. 2015;216:584-8.	Solomonides A	Stud Health Technol Inform	2015	2015/08/12			
30687852	Re-identification Risks in HIPAA Safe Harbor Data: A study of data from one environmental health study	Sweeney L, Yoo JS, Perovich L, Boronow KE, Brown P, Brody JG.	Technol Sci. 2017;2017:2017082801. Epub 2017 Aug 28.	Sweeney L	Technol Sci	2017	2019/01/29	PMC6344041	NIHMS988781	
27141525	Privacy, Security, and Patient Engagement: The Changing Health Data Governance Landscape	Holmes JH.	EGEMS (Wash DC). 2016 Mar 31;4(2):1261. doi: 10.13063/2327-9214.1261. eCollection 2016.	Holmes JH	EGEMS (Wash DC)	2016	2016/05/04	PMC4827787		10.13063/2327-9214.1261
22370452	De-identification methods for open health data: the case of the Heritage Health Prize claims dataset	El Emam K, Arbuckle L, Koru G, Eze B, Gaudette L, Neri E, Rose S, Howard J, Gluck J.	J Med Internet Res. 2012 Feb 27;14(1):e33. doi: 10.2196/jmir.2001.	El Emam K	J Med Internet Res	2012	2012/02/29	PMC3374547		10.2196/jmir.2001
29036461	Mechanisms to protect the privacy of families when using the transmission disequilibrium test in genome-	Wang M, Ji Z, Wang S, Kim J, Yang H, Jiang X, Ohno-Machado L.	Bioinformatics. 2017 Dec 1;33(23):3716-3725. doi: 10.1093/bioinformatics/btx470.	Wang M	Bioinformatics	2017	2017/10/17	PMC5860319		10.1093/bioinformatics/btx470
34011836	Reidentification of Protected Health Information: Can the Risk Be Quantified?	Higgins TL.	Crit Care Med. 2021 Jun 1;49(6):1003-1006. doi: 10.1097/CCM.0000000000004931.	Higgins TL	Crit Care Med	2021	2021/05/20			10.1097/CCM.0000000000004931
17213047	Evaluating common de-identification heuristics for personal health information	El Emam K, Jabbouri S, Sams S, Drouet Y, Power M.	J Med Internet Res. 2006 Nov 21;8(4):e28. doi: 10.2196/jmir.8.4.e28.	El Emam K	J Med Internet Res	2006	2007/01/11	PMC1794009		10.2196/jmir.8.4.e28
34139910	Protecting Privacy and Transforming COVID-19 Case Surveillance Datasets for Public Use	Lee B, Dupervil B, Deputy NP, Duck W, Soroka S, Bottichio L, Silk B, Price J, Sweeney P, Fuld J, Weber JT, Pollock D.	Public Health Rep. 2021 Sep-Oct;136(5):554-561. doi: 10.1177/00333549211026817. Epub 2021 Jun 17.	Lee B	Public Health Rep	2021	2021/06/18	PMC8216038		10.1177/00333549211026817
26739310	Anonymisation of geographical distance matrices via Lipschitz embedding	Kroll M, Schnell R.	Int J Health Geogr. 2016 Jan 7;15:1. doi: 10.1186/s12942-015-0031-7.	Kroll M	Int J Health Geogr	2016	2016/01/08	PMC4704375		10.1186/s12942-015-0031-7
32048474	What does coal mine dust lung disease look like? A radiological review following re-identification in Queer	McBean R, Tatkovic A, Edwards R, Newbigin K.	J Med Imaging Radiat Oncol. 2020 Apr;64(2):229-235. doi: 10.1111/1754-9485.13007. Epub 2020 Feb 11.	McBean R	J Med Imaging Radiat Oncol	2020	2020/02/13			10.1111/1754-9485.13007
32022691	Evaluation of Privacy Risks of Patients' Data in China: Case Study	Gong M, Wang S, Wang L, Liu C, Wang J, Guo Q, Zheng H, Xie K, Wang C, Hui Z.	JMIR Med Inform. 2020 Feb 5;8(2):e13046. doi: 10.2196/13046.	Gong M	JMIR Med Inform	2020	2020/02/06	PMC7055805		10.2196/13046
34711808	BAMboozle removes genetic variation from human sequence data for open data sharing	Ziegenhain C, Sandberg R.	Nat Commun. 2021 Oct 28;12(1):6216. doi: 10.1038/s41467-021-26152-8.	Ziegenhain C	Nat Commun	2021	2021/10/29	PMC8553849		10.1038/s41467-021-26152-8
22345546	How anonymous is 'anonymous'? Some suggestions towards a coherent universal coding system for gen	Schmidt H, Callier S.	J Med Ethics. 2012 May;38(5):304-9. doi: 10.1136/medethics-2011-100181. Epub 2012 Feb 16.	Schmidt H	J Med Ethics	2012	2012/02/21	PMC3390742	NIHMS383298	10.1136/medethics-2011-100181
24485220	Policy recommendations for addressing privacy challenges associated with cell-based research and interv	Ogbogu U, Burningham S, Ollenberger A, Calder K, Du L, El Emam K, Hyde-Lay R, Isasi R, Joly Y, Kerr I, Malin B, McDonald M, Penney S, Piat	BMC Med Ethics. 2014 Feb 3;15:7. doi: 10.1186/1472-6939-15-7.	Ogbogu U	BMC Med Ethics	2014	2014/02/04	PMC3914710		10.1186/1472-6939-15-7
2645167	Cysteine proteases of positive strand RNA viruses and chymotrypsin-like serine proteases. A distinct prot	Gorbalenya AE, Donchenko AP, Blinov VM, Koonin EV.	FEBS Lett. 1989 Jan 30;243(2):103-14. doi: 10.1016/0014-5793(89)80109-7.	Gorbalenya AE	FEBS Lett	1989	1989/01/30			10.1016/0014-5793(89)80109-7
21190545	Caught you: threats to confidentiality due to the public release of large-scale genetic data sets	Wjst M.	BMC Med Ethics. 2010 Dec 29;11:21. doi: 10.1186/1472-6939-11-21.	Wjst M	BMC Med Ethics	2010	2010/12/31	PMC3022540		10.1186/1472-6939-11-21
28982347	Outcome measures for oral health based on clinical assessments and claims data: feasibility evaluation in	Hummel R, Bruers J, van der Galiën O, van der Sanden W, van der Heijden G.	BMC Oral Health. 2017 Oct 5;17(1):125. doi: 10.1186/s12903-017-0410-5.	Hummel R	BMC Oral Health	2017	2017/10/07	PMC5629757		10.1186/s12903-017-0410-5
21893927	Is the biggest security threat to medical information simply a lack of understanding?	Williams PA.	Stud Health Technol Inform. 2011;168:179-87.	Williams PA	Stud Health Technol Inform	2011	2011/09/07			
17544262	A computational model to protect patient data from location-based re-identification	Malin B.	Artif Intell Med. 2007 Jul;40(3):223-39. doi: 10.1016/j.artmed.2007.04.002. Epub 2007 Jun 1.	Malin B	Artif Intell Med	2007	2007/06/05			10.1016/j.artmed.2007.04.002
33625129	Sharing ICU Patient Data Responsibly Under the Society of Critical Care Medicine/European Society of Int	Thoral PJ, Peppink JM, Driessen RH, Sijbrands EJG, Kompanje EJO, Kaplan L, Bailey H, Kesecioglu J, Cecconi M, Churpek M, Clermont G, va	Crit Care Med. 2021 Jun 1;49(6):e563-e577. doi: 10.1097/CCM.0000000000004916.	Thoral PJ	Crit Care Med	2021	2021/02/24	PMC8132908		10.1097/CCM.0000000000004916
26969124	Utility of linking primary care electronic medical records with Canadian census data to study the determina	Biro S, Williamson T, Leggett JA, Barber D, Morkem R, Moore K, Belanger P, Mosley B, Janssen I.	BMC Med Inform Decis Mak. 2016 Mar 11;16:32. doi: 10.1186/s12911-016-0272-9.	Biro S	BMC Med Inform Decis Mak	2016	2016/03/13	PMC4788841		10.1186/s12911-016-0272-9

24303320	Genomes in the cloud: balancing privacy rights and the public good	Ohno-Machado L, Farcas C, Kim J, Wang S, Jiang X.	AMIA Jt Summits Transl Sci Proc. 2013 Mar 18;2013:128. eCollection 2013.	Ohno-Machado	AMIA Jt Summits Transl Sci Proc	2013	2013/12/05			
34682028	Preprocessing Pipelines including Block-Matching Convolutional Neural Network for Image Denoising to F	Pawlicki M, Choraś RS.	Entropy (Basel). 2021 Oct 3;23(10):1304. doi: 10.3390/e23101304.	Pawlicki M	Entropy (Basel)	2021	2021/10/23	PMC8534374		10.3390/e23101304
23856111	[An analysis of resistance of nosocomial infection pathogens isolated from 13 teaching hospitals in 2011]	Chen HB, Zhao CJ, Wang H, Cao B, Xu XL, Chu YZ, Hu ZD, Zhuo C, Hu BJ, Liu WE, Liao K, Zhang R, Zeng J, Wang Y, Luo YP, Wang ZW, Liu Y	Zhonghua Nei Ke Za Zhi. 2013 Mar;52(3):203-12.	Chen HB	Zhonghua Nei Ke Za Zhi	2013	2013/07/17			
34737669	Using Synthetic Data to Replace Linkage Derived Elements: A Case Study	Resnick DM, Cox CS, Mirel LB.	Health Serv Outcomes Res Methodol. 2021 Feb 3;21:389-406. doi: 10.1007/s10742-021-00241-z.	Resnick DM	Health Serv Outcomes Res Methoc	2021	2021/11/05	PMC8563018	NIHMS1670775	10.1007/s10742-021-00241-z
20385806	Anonymization of electronic medical records for validating genome-wide association studies	Loukides G, Gkoulalas-Divanis A, Malin B.	Proc Natl Acad Sci U S A. 2010 Apr 27;107(17):7898-903. doi: 10.1073/pnas.0911686107. Epub 2010 Apr 12.	Loukides G	Proc Natl Acad Sci U S A	2010	2010/04/14	PMC2867915		10.1073/pnas.0911686107
26104741	Design and implementation of a privacy preserving electronic health record linkage tool in Chicago	Kho AN, Cashy JP, Jackson KL, Pah AR, Goel S, Boehnke J, Humphries JE, Kominers SD, Hota BN, Sims SA, Malin BA, French DD, Walunas T	J Am Med Inform Assoc. 2015 Sep;22(5):1072-80. doi: 10.1093/jamia/ocv038. Epub 2015 Jun 23.	Kho AN	J Am Med Inform Assoc	2015	2015/06/25	PMC5009931		10.1093/jamia/ocv038
32974512	Application of whole genome sequencing to query a potential outbreak of Elizabethkingia anophelis in Ont	McTaggart LR, Stapleton PJ, Eshaghi A, Soares D, Brisse S, Patel SN, Kus JV.	Access Microbiol. 2019 Apr 24;1(2):e000017. doi: 10.1099/acmi.0.000017. eCollection 2019.	McTaggart LR	Access Microbiol	2019	2020/09/25	PMC7470347		10.1099/acmi.0.000017
23845533	Spread of extensively resistant VIM-2-positive ST235 Pseudomonas aeruginosa in Belarus, Kazakhstan, a	Edelstein MV, Skleenova EN, Shevchenko OV, D'souza JW, Tapalski DV, Azizov IS, Sukhorukova MV, Pavlukov RA, Kozlov RS, Toleman MA, Wa	Lancet Infect Dis. 2013 Oct;13(10):867-76. doi: 10.1016/S1473-3099(13)70168-3. Epub 2013 Jul 9.	Edelstein MV	Lancet Infect Dis	2013	2013/07/13			10.1016/S1473-3099(13)70168-3
25361833	Banana infecting fungus, Fusarium musae, is also an opportunistic human pathogen: are bananas potentia	Triest D, Stubbe D, De Cremer K, Piérard D, Detandt M, Hendrickx M.	Mycologia. 2015 Jan-Feb;107(1):46-53. doi: 10.3852/14-174. Epub 2014 Oct 31.	Triest D	Mycologia	2015	2014/11/02			10.3852/14-174
20457081	Titanic's unknown child: the critical role of the mitochondrial DNA coding region in a re-identification effort	Just RS, Loreille OM, Molto JE, Merriwether DA, Woodward SR, Matheson C, Creed J, McGrath SE, Sturk-Andreaggi K, Coble MD, Irwin JA, Ru	Forensic Sci Int Genet. 2011 Jun;5(3):231-5. doi: 10.1016/j.fsigen.2010.01.012. Epub 2010 Apr 2.	Just RS	Forensic Sci Int Genet	2011	2010/05/12			10.1016/j.fsigen.2010.01.012
10482033	Microbiological characterization and clinical significance of Corynebacterium amycolatum strains	Esteban J, Nieto E, Calvo R, Fernández-Robals R, Valero-Guillén PL, Soriano F.	Eur J Clin Microbiol Infect Dis. 1999 Jul;18(7):518-21. doi: 10.1007/s100960050336.	Esteban J	Eur J Clin Microbiol Infect Dis	1999	1999/09/11			10.1007/s100960050336
8567907	Fatal cerebral mycoses caused by the ascomycete Chaetomium strumarium	Abbott SP, Sigler L, McAleer R, McGough DA, Rinaldi MG, Mizell G.	J Clin Microbiol. 1995 Oct;33(10):2692-8. doi: 10.1128/jcm.33.10.2692-2698.1995.	Abbott SP	J Clin Microbiol	1995	1995/10/01	PMC228557		10.1128/jcm.33.10.2692-2698.1995
24778614	Sharing privacy-sensitive access to neuroimaging and genetics data: a review and preliminary validation	Sarwate AD, Plis SM, Turner JA, Arbabshirani MR, Calhoun VD.	Front Neuroinform. 2014 Apr 7;8:35. doi: 10.3389/fninf.2014.00035. eCollection 2014.	Sarwate AD	Front Neuroinform	2014	2014/04/30	PMC3985022		10.3389/fninf.2014.00035
27187500	Don't Take It Personal: European Union Legal Aspects of Procuring and Protecting Environmental Exposur	Bovenberg JA, de Hoogh K, Knoppers BM, Hveem K, Hansell AL.	Biopreserv Biobank. 2016 Jun;14(3):217-23. doi: 10.1089/bio.2016.0007. Epub 2016 May 17.	Bovenberg JA	Biopreserv Biobank	2016	2016/05/18			10.1089/bio.2016.0007
172897	Growth and differentiation in culture of leukemic leukocytes from a patient with acute myelogenous leuken	Gallagher RE, Salahuddin SZ, Hall WT, McCredie KB, Gallo RC.	Proc Natl Acad Sci U S A. 1975 Oct;72(10):4137-41. doi: 10.1073/pnas.72.10.4137.	Gallagher RE	Proc Natl Acad Sci U S A	1975	1975/10/01	PMC433154		10.1073/pnas.72.10.4137
19642051	The clinical spectrum of Exophiala jeanselmei, with a case report and in vitro antifungal susceptibility of th	Badali H, Najafzadeh MJ, van Esbroeck M, van den Enden E, Tarazooie B, Meis JF, de Hoog GS.	Med Mycol. 2010 Mar;48(2):318-27. doi: 10.1080/13693780903148353.	Badali H	Med Mycol	2010	2009/07/31			10.1080/13693780903148353
22231474	Chemical ecology of astigmatid mites LXXXVII. S-(+)-isopiperitenone: re-identification of the alarm pherom	Maruno G, Mori N, Kuwahara Y.	J Chem Ecol. 2012 Jan;38(1):36-41. doi: 10.1007/s10886-012-0059-0. Epub 2012 Jan 10.	Maruno G	J Chem Ecol	2012	2012/01/11			10.1007/s10886-012-0059-0
17156451	An unsupervised classification method for inferring original case locations from low-resolution disease ma	Brownstein JS, Cassa CA, Kohane IS, Mandl KD.	Int J Health Geogr. 2006 Dec 8;5:56. doi: 10.1186/1476-072X-5-56.	Brownstein JS	Int J Health Geogr	2006	2006/12/13	PMC1702538		10.1186/1476-072X-5-56
9237562	Applications of consensus polymerase chain reaction with subsequent electrophoretic distinction of ampli	Krause G, Oduncu F, Kaltstein A, Pusl T, Pachmann K, Gerzer R.	Electrophoresis. 1997 Jun;18(7):1098-102. doi: 10.1002/elps.1150180712.	Krause G	Electrophoresis	1997	1997/06/01			10.1002/elps.1150180712
11329923	[Confidentiality within the scope of secondary data research--approaches to a solution of the problem of d	Ihle P, Krappweis J, Schubert I.	Gesundheitswesen. 2001 Mar;63 Suppl 1:S6-12. doi: 10.1055/s-2001-12105.	Ihle P	Gesundheitswesen	2001	2001/05/02			10.1055/s-2001-12105
15691645	Period analysis of cancer patient survival in datasets from which the month of diagnosis has been remove	Brenner H, Arndt V.	Eur J Cancer. 2005 Feb;41(3):438-44. doi: 10.1016/j.ejca.2004.11.018.	Brenner H	Eur J Cancer	2005	2005/02/05			10.1016/j.ejca.2004.11.018
16916188	Prevention strategy for post dural puncture headache	Gunaydin B, Karaca G.	Acta Anaesthesiol Belg. 2006;57(2):163-5.	Gunaydin B	Acta Anaesthesiol Belg	2006	2006/08/19			
16165264	[Mammary augmentation surgery and psychology. Clinical reports]	Godefroy M, Flageul G.	Ann Chir Plast Esthet. 2005 Oct;50(5):371-7. doi: 10.1016/j.anplas.2005.07.007. Epub 2005 Sep 13.	Godefroy M	Ann Chir Plast Esthet	2005	2005/09/17			10.1016/j.anplas.2005.07.007
