# Supplementary material for: Global healthcare fairness: We should be sharing more, not less, data
Source: PLOS Digit Health. 2022 Oct 6;1(10):e0000102. doi: 10.1371/journal.pdig.0000102 (PMC9931202; doi:10.1371/journal.pdig.0000102)
Supplement: S2 Table — (PDF) [file pdig.0000102.s002.pdf]

Supplementary Table 2: Coded De-Anonymization and Re-Identification Stories

stories_id	publish_date	title	url	language	ap_syndicated	themes	media_id	media_name	media_url	Code	Personal Case?
1346498876	7/23/19 15:20	'Anonymous' data might not be so anonymous, study shows	https://www.nbcnews.com/tech/tech-news/anonymous-data-might-not-be-so-anonymous-study-shows-n1033111?cid=public-rss_20190723	en	FALSE		34663	NBC Breaking News	http://www.breakingnews.com#spider	Research	0
1557562455	3/24/20 10:24	'Track and trace' is key to containing COVID-19: how privacy can be protected	https://theconversation.com/track-and-trace-is-key-to-containing-covid-19-how-privacy-can-be-protected-134369	en	FALSE		54346	Conversation	https://theconversation.com/us	Theoretical	0
1997283044	7/21/21 17:20	A Catholic priest resigned after a news site used cell phone location data to track his phone	https://hotair.com/john-s-2/2021/07/21/a-catholic-priest-resigned-after-a-news-site-used-cell-phone-location-data-to-track-his-phone-n403847	en	FALSE	christians and christia	117	Hot Air	http://hotair.com	Case	1
1022221826	10/12/18 15:38	A DNA database with 2% of the population can be used to find almost anyone	https://qz.com/1422656/public-dna-databases-will-soon-be-able-to-find-almost-anyone/	en	FALSE		67264	Quartz	http://qz.com/#spider	Theoretical	0
2025759014	8/25/21 16:16	A secret bias in mortgage-approval algorithms is denying minority borrowers	https://www.ocregister.com/2021/08/25/a-secret-bias-in-mortgage-approval-algorithms-is-denying-minority-borrowers/	en	TRUE	finances, housing	25	Orange County Register	http://www.ocregister.com/	Theoretical	0
2025754728	8/25/21 16:16	A secret bias in mortgage-approval algorithms is denying minority borrowers	https://www.dailybulletin.com/2021/08/25/a-secret-bias-in-mortgage-approval-algorithms-is-denying-minority-borrowers/	en	TRUE	finances, housing	652989	City News - United States (Pomona)	http://www.dailybulletin.com/pomona/	Theoretical	0
2025755747	8/25/21 16:16	A secret bias in mortgage-approval algorithms is denying minority borrowers	https://www.eastbaytimes.com/2021/08/25/a-secret-bias-in-mortgage-approval-algorithms-is-denying-minority-borrowers/	en	TRUE	finances, housing	659164	Berkeley Voice	http://www.insidebayarea.com/berkeley/	Theoretical	0
2025775038	8/25/21 16:16	A secret bias in mortgage-approval algorithms is denying minority borrowers	https://www.dailynews.com/2021/08/25/a-secret-bias-in-mortgage-approval-algorithms-is-denying-minority-borrowers/	en	TRUE	finances, housing	24901	Bleacher Report	http://bleacherreport.com	Theoretical	0
2025773536	8/25/21 16:16	A secret bias in mortgage-approval algorithms is denying minority borrowers	https://www.dailybulletin.com/2021/08/25/a-secret-bias-in-mortgage-approval-algorithms-is-denying-minority-borrowers/	en	TRUE	finances, housing	652990	City News - United States (Upland)	http://www.dailybulletin.com/upland/	Theoretical	0
2025771985	8/25/21 16:16	A secret bias in mortgage-approval algorithms is denying minority borrowers	https://www.whittierdailynews.com/2021/08/25/a-secret-bias-in-mortgage-approval-algorithms-is-denying-minority-borrowers/	en	TRUE	finances, housing	28067	Whittier Daily News	http://www.whittierdailynews.com/	Theoretical	0
2025770949	8/25/21 16:16	A secret bias in mortgage-approval algorithms is denying minority borrowers	https://www.redlandsdailyfacts.com/2021/08/25/a-secret-bias-in-mortgage-approval-algorithms-is-denying-minority-borrowers/	en	TRUE	finances, housing	27978	Redlands Daily Facts	http://www.redlandsdailyfacts.com/	Theoretical	0
2025770957	8/25/21 16:16	A secret bias in mortgage-approval algorithms is denying minority borrowers	https://www.sgvtribune.com/2021/08/25/a-secret-bias-in-mortgage-approval-algorithms-is-denying-minority-borrowers/	en	TRUE	finances, housing	28066	SGVTribune.com	http://www.sgvtribune.com/	Theoretical	0
2025770548	8/25/21 16:16	A secret bias in mortgage-approval algorithms is denying minority borrowers	https://www.presstelegram.com/2021/08/25/a-secret-bias-in-mortgage-approval-algorithms-is-denying-minority-borrowers/	en	TRUE	finances, housing	27911	Press-Telegram	http://www.presstelegram.com/	Theoretical	0
2025768884	8/25/21 16:16	A secret bias in mortgage-approval algorithms is denying minority borrowers	https://www.pe.com/2021/08/25/a-secret-bias-in-mortgage-approval-algorithms-is-denying-minority-borrowers/	en	TRUE	finances, housing	63	The Press-Enterprise	http://www.pe.com/	Theoretical	0
2025766141	8/25/21 16:16	A secret bias in mortgage-approval algorithms is denying minority borrowers	https://www.pasadenastarnews.com/2021/08/25/a-secret-bias-in-mortgage-approval-algorithms-is-denying-minority-borrowers/	en	TRUE	finances, housing	27963	Pasadena Star-News	http://www.pasadenastarnews.com/	Theoretical	0
2025780535	8/25/21 16:16	A secret bias in mortgage-approval algorithms is denying minority borrowers	https://www.sbsun.com/2021/08/25/a-secret-bias-in-mortgage-approval-algorithms-is-denying-minority-borrowers/	en	TRUE	finances, housing	27992	San Bernardino County Sun	http://www.sbsun.com/	Theoretical	0
2025773253	8/25/21 16:16	A secret bias in mortgage-approval algorithms is denying minority borrowers	https://www.dailybreeze.com/2021/08/25/a-secret-bias-in-mortgage-approval-algorithms-is-denying-minority-borrowers/	en	TRUE	finances, housing	28048	The Daily Breeze	http://www.dailybreeze.com/	Theoretical	0
2025768137	8/25/21 16:16	A secret bias in mortgage-approval algorithms is denying minority borrowers	https://www.eastbaytimes.com/2021/08/25/a-secret-bias-in-mortgage-approval-algorithms-is-denying-minority-borrowers/	en	TRUE	finances, housing	659204	Concord Transcript	http://www.contracostatimes.com/concord/	Theoretical	0
2025767918	8/25/21 16:16	A secret bias in mortgage-approval algorithms is denying minority borrowers	https://www.mercurynews.com/2021/08/25/a-secret-bias-in-mortgage-approval-algorithms-is-denying-minority-borrowers/	en	TRUE	finances, housing	659244	Fremont Bulletin	http://www.mercurynews.com/fremont	Theoretical	0
2025760708	8/25/21 16:16	A secret bias in mortgage-approval algorithms is denying minority borrowers	https://www.ocregister.com/2021/08/25/a-secret-bias-in-mortgage-approval-algorithms-is-denying-minority-borrowers/	en	TRUE	finances, housing	659381	Coast Magazine	http://www.coastmagazine.com/	Theoretical	0
2025764690	8/25/21 16:16	A secret bias in mortgage-approval algorithms is denying minority borrowers	https://www.dailybulletin.com/2021/08/25/a-secret-bias-in-mortgage-approval-algorithms-is-denying-minority-borrowers/	en	TRUE	finances, housing	652988	City News - United States (Ontario)	http://www.dailybulletin.com/ontario/	Theoretical	0
2025786309	8/25/21 16:16	A secret bias in mortgage-approval algorithms is denying minority borrowers	https://www.mercurynews.com/2021/08/25/a-secret-bias-in-mortgage-approval-algorithms-is-denying-minority-borrowers/	en	TRUE	finances, housing	659497	Willow Glen Resident	http://www.mercurynews.com/willow-glen/	Theoretical	0
2025810507	8/25/21 16:16	A secret bias in mortgage-approval algorithms is denying minority borrowers	https://www.dailynews.com/2021/08/25/a-secret-bias-in-mortgage-approval-algorithms-is-denying-minority-borrowers/	en	TRUE	finances, housing	74	Los Angeles Daily News	http://www.dailynews.com/	Theoretical	0
2025791685	8/25/21 16:16	A secret bias in mortgage-approval algorithms is denying minority borrowers	https://www.mercurynews.com/2021/08/25/a-secret-bias-in-mortgage-approval-algorithms-is-denying-minority-borrowers/	en	TRUE	finances, housing	659491	Almaden Resident	http://www.mercurynews.com/almaden/	Theoretical	0
2025814611	8/25/21 16:16	A secret bias in mortgage-approval algorithms is denying minority borrowers	https://www.eastbaytimes.com/2021/08/25/a-secret-bias-in-mortgage-approval-algorithms-is-denying-minority-borrowers/	en	TRUE	finances, housing	66	Contra Costa Times	http://www.contracostatimes.com/	Theoretical	0
2025762627	8/25/21 16:16	A secret bias in mortgage-approval algorithms is denying minority borrowers	https://www.mercurynews.com/2021/08/25/a-secret-bias-in-mortgage-approval-algorithms-is-denying-minority-borrowers/	en	TRUE	finances, housing	659505	San Mateo County Times	http://www.mercurynews.com/san-mateo-county-times	Theoretical	0
2026649172	8/26/21 9:19	A secret bias in mortgage-approval algorithms is denying minority borrowers	https://www.ocregister.com/2021/08/25/a-secret-bias-in-mortgage-approval-algorithms-is-denying-minority-borrowers	en	TRUE	finances, housing	86346	lagunajournal.com	http://www.lagunajournal.com/#spider	Theoretical	0
2025819250	8/25/21 16:16	A secret bias in mortgage-approval algorithms is denying minority borrowers	https://www.eastbaytimes.com/2021/08/25/a-secret-bias-in-mortgage-approval-algorithms-is-denying-minority-borrowers/	en	TRUE	finances, housing	659390	Oakley News	http://www.contracostatimes.com/oakley/	Theoretical	0
2025838872	8/25/21 16:16	A secret bias in mortgage-approval algorithms is denying minority borrowers	https://www.mercurynews.com/2021/08/25/a-secret-bias-in-mortgage-approval-algorithms-is-denying-minority-borrowers/	en	TRUE	finances, housing	35	San Jose Mercury News	http://www.mercurynews.com/	Theoretical	0
1560998658	3/27/20 7:38	A webcomic explainer on how the census deals with digital privacy	https://boingboing.net/2020/03/27/a-webcomic-explainer-on-how-th.html	en	FALSE		663527	San Antonio News	http://sanantonionews.com/	Theoretical	0
1629438332	6/8/20 20:00	ACLU wants LA to put the brakes on scooter tracking program	https://www.ocregister.com/2020/06/08/aclu-wants-la-to-put-the-brakes-on-scooter-tracking-program/	en	FALSE		25	Orange County Register	http://www.ocregister.com/	Theoretical	0
1629438980	6/8/20 20:00	ACLU wants LA to put the brakes on scooter tracking program	https://www.presstelegram.com/2020/06/08/aclu-wants-la-to-put-the-brakes-on-scooter-tracking-program/	en	FALSE		27911	Press-Telegram	http://www.presstelegram.com/	Theoretical	0
1629441345	6/8/20 20:00	ACLU wants LA to put the brakes on scooter tracking program	https://www.dailybulletin.com/2020/06/08/aclu-wants-la-to-put-the-brakes-on-scooter-tracking-program/	en	FALSE		652990	City News - United States (Upland)	http://www.dailybulletin.com/upland/	Theoretical	0
1629441601	6/8/20 20:00	ACLU wants LA to put the brakes on scooter tracking program	https://www.dailynews.com/2020/06/08/aclu-wants-la-to-put-the-brakes-on-scooter-tracking-program/	en	FALSE		24901	Bleacher Report	http://bleacherreport.com	Theoretical	0
1629441401	6/8/20 20:00	ACLU wants LA to put the brakes on scooter tracking program	https://www.pasadenastarnews.com/2020/06/08/aclu-wants-la-to-put-the-brakes-on-scooter-tracking-program/	en	FALSE		27963	Pasadena Star-News	http://www.pasadenastarnews.com/	Theoretical	0
1629443047	6/8/20 20:00	ACLU wants LA to put the brakes on scooter tracking program	https://www.dailybulletin.com/2020/06/08/aclu-wants-la-to-put-the-brakes-on-scooter-tracking-program/	en	FALSE		652988	City News - United States (Ontario)	http://www.dailybulletin.com/ontario/	Theoretical	0
1629443632	6/8/20 20:00	ACLU wants LA to put the brakes on scooter tracking program	https://www.sgvtribune.com/2020/06/08/aclu-wants-la-to-put-the-brakes-on-scooter-tracking-program/	en	FALSE		28066	SGVTribune.com	http://www.sgvtribune.com/	Theoretical	0

1629444727	6/8/20 20:00	ACLU wants LA to put the brakes on scooter tracking program	https://www.whittierdailynews.com/2020/06/08/aclu-wants-la-to-put-the-brakes-on-scooter-tracking-program/	en	FALSE		28067	Whittier Daily News	http://www.whittierdailynews.com/	Theoretical	0
1629444950	6/8/20 20:00	ACLU wants LA to put the brakes on scooter tracking program	https://www.dailybulletin.com/2020/06/08/aclu-wants-la-to-put-the-brakes-on-scooter-tracking-program/	en	FALSE		652989	City News - United States (Pomona)	http://www.dailybulletin.com/pomona/	Theoretical	0
1629445820	6/8/20 20:00	ACLU wants LA to put the brakes on scooter tracking program	https://www.dailybreeze.com/2020/06/08/aclu-wants-la-to-put-the-brakes-on-scooter-tracking-program/	en	FALSE		28048	The Daily Breeze	http://www.dailybreeze.com/	Theoretical	0
1629446824	6/8/20 20:00	ACLU wants LA to put the brakes on scooter tracking program	https://www.dailybulletin.com/2020/06/08/aclu-wants-la-to-put-the-brakes-on-scooter-tracking-program/	en	FALSE		27951	DailyBulletin.com	http://www.dailybulletin.com/	Theoretical	0
1629446737	6/8/20 20:00	ACLU wants LA to put the brakes on scooter tracking program	https://www.dailynews.com/2020/06/08/aclu-wants-la-to-put-the-brakes-on-scooter-tracking-program/	en	FALSE		74	Los Angeles Daily News	http://www.dailynews.com/	Theoretical	0
1393593177	2/16/19 0:00	After you spit into a tube for a DNA test like 23andMe, experts say you shouldn't assume you	https://www.businessinsider.com/privacy-security-risks-genetic-testing-23andme-ancestry-dna-2019-2#comments	en	FALSE		18710	Business Insider	http://www.businessinsider.com	Research	0
667558993	8/1/17 15:58	Amazon Takes Privacy Stand by Slapping Down Blu for Sending Personal Data to China	http://observer.com/2017/08/amazon-kryptowire-blu-cubot/	en	FALSE		1063	Politicker NJ	http://www.politickernj.com/	Theoretical	0
1050201241	8/1/17 15:58	Amazon Takes Privacy Stand by Slapping Down Blu for Sending Personal Data to China	http://observer.com/2017/08/amazon-kryptowire-blu-cubot/	en	FALSE		21091	observer.com	http://www.observer.com	Theoretical	0
607543662	4/11/17 10:20	An Algorithm That Hides Your Online Tracks With Random Footsteps	http://feedproxy.google.com/~r/TheAtlantic/~3/2CQLH_GMnCI/	en	FALSE		1110	Atlantic	http://www.theatlantic.com/	Theoretical	0
1347725158	7/24/19 14:56	Anonymized data isn't actually keeping your identity anonymous, researchers find	https://www.google.com/url?rct=j&sa=t&url=https://www.mic.com/p/anonymized-data-isnt-actually-keeping-your-identity-anonymous-researcher	en	FALSE		109076	Mic	http://mic.com/	Research	0
1941939256	5/25/21 14:12	Best browser for privacy 2021: Secure your web browsing	https://www.zdnet.com/article/best-browser-for-privacy/#ftag=RSSbaffb68	en	FALSE	privacy, computers an	39330	zdnet	http://news.zdnet.com/	Research	0
1576218059	4/12/20 9:00	Big Data Could Undermine the Covid-19 Response	https://www.wired.com/story/big-data-could-undermine-the-covid-19-response	en	FALSE		39849	wired	http://www.wired.com/	Research	0
1605708376	5/13/20 7:02	Big Tech offers a way to track COVID-19 with cell phones	http://dallasposttrib.com/big-tech-offers-a-way-to-track-covid-19-with-cell-phones/	en	FALSE		104386	Dallas Post Tribune | Serving the Blac	http://www.dallasposttrib.com	Theoretical	0
919962666	7/3/18 15:53	Blockchain and GDPR — Frenemies?	http://feeds.feedblitz.com/-/556179446/0/law/legal-news/	en	FALSE		112662	thelegalintelligencer.com	http://thelegalintelligencer.com/	Theoretical	0
1996963197	7/21/21 12:57	Catholic priest quits after “anonymized” data revealed alleged use of Grindr	https://arstechnica.com/?p=1781933	en	FALSE	privacy, christians and	18351	arstechnica.com	http://arstechnica.com	Case	1
521848552	10/5/16 2:29	CloudFlare shows Tor users the way out of CAPTCHA hell	http://go.theregister.com/feed/www.theregister.co.uk/2016/10/05/cloudflare_tor/	en	FALSE		5532	The Register	http://www.theregister.co.uk/	Theoretical	0
1692421393	8/25/20 9:00	Column: Billboards that follow you? It's not sci-fi. They're already here	http://feeds.latimes.com/~r/latimes/business/~3/4Y2XT-TrZXk/column-clear-channel-billboards-privacy	en	FALSE		6	LA Times	http://www.latimes.com/	Theoretical	0
1692722643	8/25/20 16:44	Column: Billboards that follow you? It’s not sci-fi. They’re already here [Los Angeles Times]	https://www.dailyrepublic.com/all-dr-news/wires/column-billboards-that-follow-you-its-not-sci-fi-theyre-already-here-los-angeles-times-2/	en	FALSE		27866	Daily Republic | Solano County's Brea	http://www.dailyrepublic.com/	Theoretical	0
566164912	1/11/17 9:00	Companies are making billions off your medical data — and you won't get a cent of it	http://mic.com/articles/165093/companies-are-making-billions-off-your-medical-data-and-you-wont-get-a-cent-of-it	en	FALSE		109076	Mic	http://mic.com/	Theoretical	0
1218216824	3/8/19 10:13	Connected Cars: Security And Privacy Are Both Trust Hurdles	https://www.forbes.com/sites/taylorarmerding/2019/03/08/connected-cars-security-and-privacy-are-both-trust-hurdles/	en	FALSE		1104	Forbes	http://www.forbes.com/	Theoretical	0
1908005810	4/20/21 20:01	Conservatives Should Support the College Transparency Act	https://townhall.com/columnists/janerobbins/2021/04/21/college-transparency-act-n2588266	en	FALSE		19763	townhall.com	http://townhall.com	Theoretical	0
1545015146	3/11/20 10:50	Coronavirus In Tennessee: Most Cases Are In Williamson County	https://www.wknofm.org/post/coronavirus-tennessee-most-cases-are-williamson-county	en	FALSE		90047	wknofm.org	http://wknofm.org/#spider	Theoretical	0
1578236864	4/15/20 0:15	Coronavirus tracing tech policy 'more significant' than the war on encryption	https://www.zdnet.com/article/coronavirus-tracing-tech-policy-more-significant-than-the-war-on-encryption/#ftag=RSSbaffb68	en	FALSE		39330	zdnet	http://news.zdnet.com/	Research	0
1920349411	5/3/21 16:48	Data Privacy And Cryptography Experts File Brief In Support Of Census Bureau	https://www.thestreet.com/press-releases/data-privacy-and-cryptography-experts-file-brief-in-support-of-census-bureau-15643134	en	FALSE		21619	The Street	http://www.thestreet.com	Theoretical	0
705798451	10/13/17 8:17	Dear America, you can't steal a personality: GDPR godfather talks privacy with <i>El Reg</i>	http://go.theregister.com/feed/www.theregister.co.uk/2017/10/13/jan_philipp_albrecht_on_privacy/	en	FALSE		5532	The Register	http://www.theregister.co.uk/	Theoretical	0
1025509316	10/15/18 19:00	DNA testing companies put everyone’s privacy at risk	https://www.salon.com/2018/10/15/dna-testing-companies-puts-everyones-privacy-at-risk/	en	FALSE		1757	Salon	http://www.salon.com/	Theoretical	0
845646595	4/10/18 1:01	Don't be so shocked at spies tracking your phone	http://www.heralddemocrat.com/opinion/20180410/don8217t-be-so-shocked-at-spies-tracking-your-phone?rssfeed=true	en	FALSE		27089	heralddemocrat.com	http://heralddemocrat.com	Research	0
1928396558	5/11/21 19:01	Elderly caretech platform Birdie gets $11.5M Series A led by Index	https://techcrunch.com/2021/05/11/elderly-caretech-platform-birdie-gets-11-5m-series-a-led-by-index/	en	FALSE		4438	TechCrunch	http://techcrunch.com	Theoretical	0
1928483059	5/11/21 19:01	Elderly caretech platform Birdie gets $11.5M Series A led by Index	https://api.follow.it/track-rss-story-click/v2/U5jZ60AhOyf49TMAeZPkrJVjH6qdYdAk?urllink=https%253A%252F%252Ftechcrunch.com%252F20	en	FALSE	finances	34587	pasadenaindependent.com	http://www.pasadenaindependent.com#spider	Theoretical	0
1928484088	5/11/21 19:01	Elderly caretech platform Birdie gets $11.5M Series A led by Index	https://api.follow.it/track-rss-story-click/v2/U5jZ60AhOyf49TMAeZPkrJVjH6qdYdAk?urllink=https%253A%252F%252Ftechcrunch.com%252F20	en	FALSE	finances	131661	morgannews.com	http://morgannews.com/	Theoretical	0
1928486232	5/11/21 19:01	Elderly caretech platform Birdie gets $11.5M Series A led by Index	https://api.follow.it/track-rss-story-click/v2/U5jZ60AhOyf49TMAeZPkrJVjH6qdYdAk?urllink=https%253A%252F%252Ftechcrunch.com%252F20	en	FALSE	finances	34586	arcadiaweekly.com	http://www.arcadiaweekly.com#spider	Theoretical	0
1928509244	5/11/21 19:01	Elderly caretech platform Birdie gets $11.5M Series A led by Index	https://api.follow.it/track-rss-story-click/v2/U5jZ60AhOyf49TMAeZPkrJVjH6qdYdAk?urllink=https%253A%252F%252Ftechcrunch.com%252F20	en	FALSE	finances	31448	monroviaweekly.com	http://www.monroviaweekly.com#spider	Theoretical	0
1620027480	5/29/20 7:50	Ethereum Significantly Less Private Than Bitcoin, New Research Shows	https://cointelegraph.com/news/ethereum-significantly-less-private-than-bitcoin-new-research-shows	en	FALSE		360949	http://www.ct.com/news/advocates/	http://www.ct.com/news/advocates/	Research	0
841414929	4/5/18 16:52	Facebook spoke with hospitals about matching health data to anonymized profiles	https://www.theverge.com/2018/4/5/17203262/facebook-medical-data-sharing-plan-healthcare	en	FALSE		107634	Verge	http://theverge.com/	Theoretical	0
1420483137	10/16/19 11:32	Feds Torpedo Massive Dark Web Child Porn Site	https://www.thedailybeast.com/feds-take-down-welcome-to-video-a-massive-dark-web-child-porn-site?source=articles&via=rss	en	FALSE		1707	Daily Beast	http://www.thedailybeast.com/	Case	0
612699859	4/21/17 13:53	Form an orderly queue! Google wants your blood (and other bodily fluids). Oh and your medi	https://techcrunch.com/2017/04/21/form-an-orderly-queue-google-wants-your-blood-and-other-bodily-fluids-oh-and-your-medical-records/?ncic	en	FALSE		4438	TechCrunch	http://techcrunch.com	Theoretical	0
925995247	7/12/18 5:10	Fugitive Cop Says He’s Behind the DNC Leaks. It’s His Latest Hoax.	http://feedproxy.google.com/~r/thedailybeast/articles/~3/pAkBIRygHes/fugitive-cop-says-hes-behind-the-dnc-leaks-its-his-latest-hoax	en	FALSE		1707	Daily Beast	http://www.thedailybeast.com/	Case	1
1138797303	1/8/19 1:58	FYI: Twitter's API still spews enough metadata to reveal exactly where you lived, worked	http://go.theregister.com/feed/www.theregister.co.uk/2019/01/08/twitter_privacy_problems/	en	FALSE		5532	The Register	http://www.theregister.co.uk/	Theoretical	0
1434707219	11/1/19 19:03	Google bought Fitbit. What does that mean for your data privacy?	https://www.pbs.org/newshour/economy/making-sense/google-bought-fitbit-what-does-that-mean-for-your-data-privacy	en	FALSE		271188	PBS NewsHour	http://www.pbs.org/newshour/	Theoretical	0

1396884014	9/19/19 8:46	Google completes controversial takeover of DeepMind Health	https://techcrunch.com/2019/09/19/google-completes-controversial-takeover-of-deepmind-health/	en	FALSE		4438	TechCrunch	http://techcrunch.com	Theoretical	0	
1396954480	9/19/19 8:46	Google completes controversial takeover of DeepMind Health	https://www.specificfeeds.com/track-rss-story-click/Q3dp_Nkg6aDaXSztMMpIJOWfknnxp1pP19-cryXjmpJ8-NBGn1kZvtb0fNgR8Yg5FHYOtuMz	en	FALSE		131661	morgannews.com	http://morgannews.com/	Theoretical	0	
1384274732	9/5/19 15:21	Google releases a free/open differential privacy library	https://boingboing.net/2019/09/05/cakeism-within-reach.html	en	FALSE		663527	San Antonio News	http://sanantonionews.com/	Theoretical	0	
1177149981	1/28/19 8:05	Google’s Sidewalk Labs Plans to Package and Sell Location Data on Millions of Cellp	https://theintercept.com/2019/01/28/google-alphabet-sidewalk-labs-replica-cellphone-data/	en	FALSE		269138	Intercept	https://theintercept.com/	Theoretical	0	
893381376	6/5/18 8:44	Healthcare Data As Property Can Change Everything	https://www.forbes.com/sites/johnnosta/2018/06/05/healthcare-data-as-property-can-change-everything/	en	FALSE		1104	Forbes	http://www.forbes.com/	Theoretical	0	
1970076725	6/23/21 14:22	Hospitals are selling treasure troves of medical data — what could go wrong?	https://www.theverge.com/2021/6/23/22547397/medical-records-health-data-hospitals-research	en	FALSE	privacy, computers an	107634	Verge	http://theverge.com/	Theoretical	0	
835400394	2/1/18 16:30	How a fitness app revealed military secrets — and the new reality of data collection	https://www.vox.com/technology/2018/2/1/16945120/strava-data-tracking-privacy-military-bases	en	FALSE		104828	Vox	http://www.vox.com	Theoretical	0	
608487443	4/13/17 1:03	How social media data can improve people’s lives - if used responsibly	http://theconversation.com/how-social-media-data-can-improve-peoples-lives-if-used-responsibly-75367	en	FALSE		54346	Conversation	https://theconversation.com/us	Theoretical	0	
1959454011	6/13/21 1:00	How your medical data can save lives | Letters	https://www.theguardian.com/commentisfree/2021/jun/13/how-medical-data-can-save-lives-observer-letters	en	FALSE	medicine and health, c	1751	Guardian US	https://www.theguardian.com/us	Theoretical	0	
1290392270	5/22/19 13:50	In less than one second, a malicious web-page can uniquely fingerprint an Iphone, Pixel 2 or	https://boingboing.net/2019/05/22/unique-device-fingerprints.html	en	FALSE		663527	San Antonio News	http://sanantonionews.com/	Research	0	
1990117400	7/14/21 9:00	Inside the Industry That Unmasks People At Scale	https://www.vice.com/en_us/article/epnmvz/industry-unmasks-at-scale-maid-to-pii	en	FALSE	politics and governme	300613	VICE	http://www.vice.com/en_us	Case	0	Reporting
1420483956	10/16/19 12:06	IRS Followed Bitcoin Transactions, Resulting In Takedown Of The Largest Child Exploitation	https://www.forbes.com/sites/kellyphillipserb/2019/10/16/irs-followed-bitcoin-transactions-resulting-in-takedown-of-the-largest-child-exploitation	en	FALSE		1104	Forbes	http://www.forbes.com/	Case	0	Reporting
1346575846	7/23/19 18:27	It's not that hard to unmask real people in anonymous data, researchers warn - CNET	https://www.cnet.com/news/its-not-that-hard-to-unmask-real-people-in-anonymous-data-researchers-warn/#ftag=CADf328eec	en	FALSE		4415	CNET	http://cnet.com	Research	0	
1544224025	3/10/20 16:01	Knox County health officials: We won't hide it if a coronavirus case is confirmed here	http://rssfeeds.knoxnews.com/~/619655816/0/knoxville/home/~Knox-County-health-officials-We-wont-hide-it-if-a-coronavirus-case-is-confirmed	en	FALSE		89	The Knoxville News Sentinel	http://www.knoxnews.com/	Theoretical	0	
1544226463	3/10/20 16:01	Knox County health officials: We won't hide it if a coronavirus case is confirmed here	http://rssfeeds.knoxnews.com/~/619655816/0/knoxville/home/~Knox-County-health-officials-We-wont-hide-it-if-a-coronavirus-case-is-confirmed	en	FALSE		607964	Shopper News	https://www.knoxnews.com/shopper-news/	Theoretical	0	
1899711947	4/7/21 8:30	LiveRamp Enters into Strategic Partnership with Google Cloud to Enable Identity in the Cloud	http://www.businesswire.com/news/home/20210407005290/en/LiveRamp-Enters-into-Strategic-Partnership-with-Google-Cloud-to-Enable-Identi	en	FALSE		659485	Western Edition	http://www.thewesternedition.com/	Theoretical	0	
1571477410	4/7/20 12:15	Location Tracking to Fight Coronavirus Is Dangerous and Possibly Pointless	https://gizmodo.com/location-tracking-to-fight-coronavirus-is-dangerous-and-1842726733	en	FALSE		4469	Gizmodo	http://gizmodo.com	Theoretical	0	
1193138147	2/12/19 17:00	Medical database privacy	https://techcrunch.com/2019/02/12/medical-database-privacy/	en	FALSE		4438	TechCrunch	http://techcrunch.com	Research	0	
1828210820	1/19/21 11:29	Mirador Analytics Partners With Datavant To Create Step Change In Industry's Approach To I	https://www.thestreet.com/press-releases/mirador-analytics-partners-with-datavant-to-create-step-change-in-industry-s-approach-to-hipaa-de-i	en	FALSE		21619	The Street	http://www.thestreet.com	Theoretical	0	
1590190804	4/27/20 9:53	Mobile phone data is useful in coronavirus battle. But are people protected enough?	https://theconversation.com/mobile-phone-data-is-useful-in-coronavirus-battle-but-are-people-protected-enough-136404	en	FALSE		54346	Conversation	https://theconversation.com/us	Theoretical	0	
1999208510	7/23/21 16:31	Msgr. Burrill’s Resignation and the Surveillance Age: 5 Points to Consider	http://feedproxy.google.com/~r/NCRegisterDailyBlog/~3/peuT0j128oc/msgr-burrill-s-resignation-and-the-surveillance-age-5-points-to-consider	en	FALSE	homosexuality	23290	National Catholic Register	http://www.ncregister.com	Case	1	Catholic p
2003217604	7/28/21 4:00	New Catholic website 'The Pillar' operates on shaky journalistic foundation	https://www.ncronline.org/news/new-catholic-website-pillar-operates-shaky-journalistic-foundation	en	FALSE	homosexuality, ethics,	33885	ncronline.org	http://ncronline.org#spider	Case	1	Catholic p
749342859	12/17/17 20:01	No hack needed: Anonymisation beaten with a dash of SQL	http://go.theregister.com/feed/www.theregister.co.uk/2017/12/18/no_hack_needed_anonymisation_beaten_with_a_dash_of_sql/	en	FALSE		5532	The Register	http://www.theregister.co.uk/	Research	0	
1833435051	2/15/17 0:00	Not Your Grandpa’s Payphone | The Indypendent	https://indypendent.org/2017/02/not-your-grandpas-payphone/	en	FALSE		74548	indypendent.org	http://www.indypendent.org/#spider	Theoretical	0	
1486081445	12/21/19 13:00	Opinion | Where Even the Children Are Being Tracked - The New York Times	https://www.nytimes.com/interactive/2019/12/21/opinion/pasadena-smartphone-spying.html	en	FALSE		1	New York Times	http://nytimes.com	Case	1	NYT inves
1962416424	6/16/21 1:38	Patient privacy in the big data era - Boston Children's Answers	https://answers.childrenshospital.org/patient-privacy-big-data/	en	FALSE	privacy, tests and testi	658824	State News - United States - Massac	http://www.massachusetts.statenews.net/	Theoretical	0	
1558355077	3/25/20 5:31	Privacy And Data Innovation Professionals Discuss Future Of Personal Marketing	https://www.thestreet.com/press-releases/privacy-and-data-innovation-professionals-discuss-future-of-personal-marketing-15274852	en	FALSE		21619	The Street	http://www.thestreet.com	Theoretical	0	
884890903	11/17/17 0:00	Privacy breaches in University file system affect 200 people	https://www.stanforddaily.com/2017/11/17/privacy-breaches-in-university-file-system-affect-200-people/	en	FALSE		30255	Stanford Daily	http://www.stanforddaily.com#spider	Case	0	
1685334845	8/17/20 7:00	Privacy Issues Still At Play With Many Contact-Tracing Tools	https://www.forbes.com/sites/forbestechcouncil/2020/08/17/privacy-issues-still-at-play-with-many-contact-tracing-tools/	en	FALSE		1104	Forbes	http://www.forbes.com/	Research	0	
1367291490	8/17/19 9:00	Privacy researchers devise a noise-exploitation attack that defeats dynamic anonymity	https://techcrunch.com/2019/08/17/privacy-researchers-devise-a-noise-exploitation-attack-that-defeats-dynamic-anonymity/	en	FALSE		4438	TechCrunch	http://techcrunch.com	Research	0	
667554455	8/1/17 15:39	Private Browsing Really Anonymous? Incognito Mode Histories Exposed By Researchers' Fa	http://www.ibtimes.com/private-browsing-really-anonymous-incognito-mode-histories-exposed-researchers-fake-2573095	en	FALSE		41550	IBTimes	http://www.ibtimes.com/	Case	0	
1968615518	6/22/21 9:15	Prognos Health And Mirador Analytics Form Strategic Partnership To Streamline Expert Dete	https://www.thestreet.com/press-releases/prognos-health-and-mirador-analytics-form-strategic-partnership-to-streamline-expert-determination-t	en	FALSE	privacy, tests and testi	21619	The Street	http://www.thestreet.com	Theoretical	0	
1711198218	9/14/20 16:52	Race-based COVID-19 data may be used to discriminate against racialized communities	https://theconversation.com/race-based-covid-19-data-may-be-used-to-discriminate-against-racialized-communities-138372	en	FALSE		54346	Conversation	https://theconversation.com/us	Theoretical	0	
1272335723	5/2/19 13:06	Report: Apps to Help Fight Depression and Quit Smoking Sell User Data to Facebook, Goog	http://feedproxy.google.com/~r/breitbart/~3/W0OK0BabTVc/	en	FALSE		658595	Breitbart - United States - Texas	http://www.breitbart.com/texas/	Theoretical	0	
1272335250	5/2/19 13:06	Report: Apps to Help Fight Depression and Quit Smoking Sell User Data to Facebook, Goog	http://feedproxy.google.com/~r/breitbart/~3/W0OK0BabTVc/	en	FALSE		19334	Breitbart	http://www.breitbart.com	Theoretical	0	
1346458032	7/23/19 11:44	Researchers Reveal That Anonymized Data Is Easy To Reverse Engineer	https://gizmodo.com/researchers-reveal-that-anonymized-data-is-easy-to-reve-1836629166	en	FALSE		4469	Gizmodo	http://gizmodo.com	Research	0	
1347024673	7/24/19 6:30	Researchers spotlight the lie of ‘anonymous’ data	https://techcrunch.com/2019/07/24/researchers-spotlight-the-lie-of-anonymous-data/	en	FALSE		4438	TechCrunch	http://techcrunch.com	Research	0	
1382978071	9/4/19 10:06	Researchers Use Big Data And AI To Remove Legal Confidentiality	https://www.forbes.com/sites/simonchandler/2019/09/04/researchers-use-big-data-and-ai-to-remove-legal-confidentiality/	en	FALSE		1104	Forbes	http://www.forbes.com/	Research	0	

1234103437	3/27/19 13:16	Rethinking Privacy For The AI Era	https://www.forbes.com/sites/insights-intelai/2019/03/27/rethinking-privacy-for-the-ai-era/	en	FALSE		1104	Forbes	http://www.forbes.com/	Theoretical	0	
1591779434	3/9/20 0:00	Say Hello to Your 1.23-Ounce Big Brother: Fitbits Could Be Used to Track Viruses Like Covid	https://pjmedia.com/news-and-politics/paula-bolyard/2020/03/09/your-fitbit-could-be-used-to-monitor-the-spread-of-coronavirus-is-that-really-v	en	FALSE		19082	PJ Media	http://pjmedia.com	Theoretical	0	
863269194	5/1/18 8:30	Scott Pruitt's new 'secret science' proposal is the wrong way to increase transparency	https://www.theverge.com/2018/5/1/17304298/epa-science-transparency-rule-scott-pruitt-data-sharing	en	FALSE		107634	Verge	http://theverge.com/	Theoretical	0	
1592515239	4/29/20 12:12	Security experts warn: Don't let contact-tracing app lead to surveillance	https://www.zdnet.com/article/security-experts-warn-dont-let-contact-tracing-app-lead-to-surveillance/#ftag=RSSbaffb68	en	FALSE		39330	zdnet	http://news.zdnet.com/	Theoretical	0	
1659155202	5/7/20 11:55	Security experts warn: Don't let contact-tracing app lead to surveillance | ZDNet	https://www.zdnet.com/article/security-experts-warn-dont-let-contact-tracing-app-lead-to-surveillance/?ftag=COS-05-10aaa0h&utm_campaign=	en	FALSE		39330	zdnet	http://news.zdnet.com/	Theoretical	0	
1649691292	7/1/20 5:09	Smart cities will track our every move. We will need to keep them in check	https://www.zdnet.com/article/smart-cities-will-track-our-every-move-we-will-need-to-keep-them-in-check/#ftag=RSSbaffb68	en	FALSE		39330	zdnet	http://news.zdnet.com/	Theoretical	0	
1263894329	4/22/19 14:02	Smoking and depression apps are selling your data to Google and Facebook, study finds	https://www.washingtonpost.com/business/smoking-and-depression-apps-are-selling-your-data-to-google-and-facebook-study-finds/2019/04/2	en	FALSE		2	Washington Post	http://washingtonpost.com	Theoretical	0	
1269216050	4/29/19 8:00	Smoking, depression apps sell your data to Google and Facebook, study finds	https://www.denverpost.com/2019/04/29/smoking-depression-apps-data-google-facebook/	en	FALSE		390346	Latest news, sports, weather from De	http://feeds.denverpost.com/dp-news-breaking	Theoretical	0	
2017260233	8/12/21 5:50	States Are Suing the Census Bureau Over Its Attempts to Make Data More Private	https://slate.com/technology/2021/08/census-bureau-differential-privacy-lawsuit.html?via=rss	en	FALSE	privacy, crime and crir	19643	Slate.com	http://www.slate.com	Research	0	
889561246	9/4/17 0:00	Study finds new way genome privacy can be breached	http://www.sandiegouniontribune.com/business/biotech/sd-me-privacy-genomes-20170904-story.html	en	FALSE		268225	San Diego Union Tribune	http://sandiegouniontribune.com/	Research	0	
1279342126	5/10/19 9:10	The battle over Trump's tax returns, explained	https://www.vox.com/policy-and-politics/2019/5/10/18537282/trump-tax-returns-congress-irs	en	FALSE		104828	Vox	http://www.vox.com	Case	1	Trump tax
959881886	8/21/18 12:46	The company that analyzed your DNA just sold the results to someone else. Really, what are	http://www.ksl.com/ad_logger/ad_logger.php?location=https://www.deseretnews.com/article/900028783/the-company-that-analyzed-your-dna-ju	en	FALSE		69357	ksl.com	http://www.ksl.com/#spider	Theoretical	0	
1506029382	1/27/20 11:04	The cost of Avast's Free Antivirus: Companies can spy on your clicks	http://feeds.mashable.com/~r/Mashable/~3/WJldEmWYI_U/	en	FALSE		5527	Mashable!	http://mashable.com	Theoretical	0	
522727935	10/6/16 19:38	The FCC wants ISPs to get permission before sharing your data	https://www.engadget.com/2016/10/06/fcc-internet-privacy-data-rules-proposal-october/	en	FALSE		41231	engadget	http://www.engadget.com/	Theoretical	0	
1842776176	2/4/21 14:16	The Genome You Sent to 23andMe Now Belongs to Richard Branson, Too	https://www.vice.com/en_us/article/wx8kg4/the-genome-you-sent-to-23andme-now-belongs-to-richard-branson-too	en	FALSE		300613	VICE	http://www.vice.com/en_us	Theoretical	0	
1004188485	1/24/17 5:00	The Hidden Global Trade in Patient Medical Data | YaleGlobal Online	https://yaleglobal.yale.edu/content/hidden-global-trade-patient-medical-data?utm_source=ListServe&utm_campaign=07f9a8e34d-EMAIL_CAMP	en	FALSE		32406	Yale Global	http://yaleglobal.yale.edu#spider	Theoretical	0	
2025476614	8/25/21 6:57	The secret bias hidden in mortgage-approval algorithms	http://www.therepublic.com/2021/08/25/us-mortgage-approval-algorithms/	en	FALSE	finances, housing	21258	m.therepublic.com	http://m.therepublic.com	Theoretical	0	
2025472059	8/25/21 6:57	The secret bias hidden in mortgage-approval algorithms	http://www.dailyjournal.net/2021/08/25/us-mortgage-approval-algorithms/	en	FALSE	finances, housing	74310	dailyjournal.net	http://dailyjournal.net/#spider	Theoretical	0	
2025470993	8/24/21 18:56	The secret bias hidden in mortgage-approval algorithms	https://mynorthwest.com/3107974/the-secret-bias-hidden-in-mortgage-approval-algorithms/	en	FALSE	finances, housing	56743	mynorthwest.com	http://mynorthwest.com/	Theoretical	0	
2025462407	8/25/21 2:57	The secret bias hidden in mortgage-approval algorithms	http://www.mynews13.com/fl/orlando/ap-online/2021/08/25/the-secret-bias-hidden-in-mortgage-approval-algorithms	en	FALSE	finances, housing	87980	mynews13.com	http://mynews13.com/#spider	Theoretical	0	
2025485120	8/25/21 2:57	The secret bias hidden in mortgage-approval algorithms	http://www.baynews9.com/fl/tampa/ap-online/2021/08/25/the-secret-bias-hidden-in-mortgage-approval-algorithms	en	FALSE	finances, housing	25893	baynews9.com	http://www.baynews9.com	Theoretical	0	
2025483454	8/25/21 6:56	The secret bias hidden in mortgage-approval algorithms	https://ktar.com/story/4652898/the-secret-bias-hidden-in-mortgage-approval-algorithms/	en	FALSE	finances, housing	25379	ktar.com	http://ktar.com	Theoretical	0	
2025467857	8/25/21 6:57	The secret bias hidden in mortgage-approval algorithms	http://www.artesianews.com/2004852/the-secret-bias-hidden-in-mortgage-approval-algorithms.html	en	FALSE	housing	309726	artesianews.com	http://artesianews.com/	Theoretical	0	
2025502192	8/25/21 1:56	The secret bias hidden in mortgage-approval algorithms	https://www.marketbeat.com/articles/the-secret-bias-hidden-in-mortgage-approval-algorithms-2021-08-25/	en	FALSE	housing	660015	Macon Daily	http://www.macondaily.com/	Theoretical	0	
2025548044	8/25/21 9:25	The secret bias hidden in mortgage-approval algorithms	https://sentinelcolorado.com/news/nation-world/nation/the-secret-bias-hidden-in-mortgage-approval-algorithms/	en	FALSE	finances, housing	87321	aurorasentinel.com	http://www.aurorasentinel.com/#spider	Theoretical	0	
2025597108	8/25/21 9:25	The secret bias hidden in mortgage-approval algorithms	https://sentinelcolorado.com/news/nation-world/nation/the-secret-bias-hidden-in-mortgage-approval-algorithms/	en	FALSE	finances, housing	659573	Aurora Magazine	http://www.aurorasentinel.com/aurora-magazine/	Theoretical	0	
2025747972	8/25/21 12:03	The secret bias hidden in mortgage-approval algorithms	https://www.beaumontenterprise.com/news/article/The-secret-bias-hidden-in-mortgage-approval-16410286.php	en	FALSE	finances, housing	663445	Hardin County News	http://www.beaumontenterprise.com/hardincountynews/	Theoretical	0	
2025719392	8/25/21 12:03	The secret bias hidden in mortgage-approval algorithms	https://www.fltimes.com/business/national/the-secret-bias-hidden-in-mortgage-approval-algorithms/article_0137bbab-a06f-5f73-a2e5-94071fdc	en	FALSE	finances, housing	28216	Finger Lakes Times: Finger Lakes Tin	http://www.fltimes.com/	Theoretical	0	
2025667552	8/25/21 13:08	The secret bias hidden in mortgage-approval algorithms	http://www.winknews.com/2021/08/25/the-secret-bias-hidden-in-mortgage-approval-algorithms/	en	TRUE	finances, housing	80060	winknews.com	http://www.winknews.com/#spider	Theoretical	0	
2025573414	8/25/21 6:56	The secret bias hidden in mortgage-approval algorithms	https://www.timesunion.com/business/article/The-secret-bias-hidden-in-mortgage-approval-16410286.php	en	FALSE	finances, housing	20821	blog.timesunion.com	http://blog.timesunion.com	Theoretical	0	
2025483191	8/25/21 6:56	The secret bias hidden in mortgage-approval algorithms	https://federalnewsnetwork.com/government-news/2021/08/the-secret-bias-hidden-in-mortgage-approval-algorithms/	en	FALSE	finances, housing	22565	federalnewsradio.com	http://www.federalnewsradio.com	Theoretical	0	
2025460910	8/25/21 6:57	The secret bias hidden in mortgage-approval algorithms	http://www.bcdemocrat.com/2021/08/25/us-mortgage-approval-algorithms/	en	FALSE	finances, housing	468106	bcdemocrat.com	http://bcdemocrat.com/	Theoretical	0	
2025874110	8/25/21 12:30	The Secret Bias Hidden in Mortgage-Approval Algorithms	https://businessjournaldaily.com/the-secret-bias-hidden-in-mortgage-approval-algorithms/	en	FALSE	housing	189893	businessjournaldaily.com	http://businessjournaldaily.com/	Theoretical	0	
2025848506	8/25/21 13:00	The secret bias hidden in mortgage-approval algorithms	https://decaturdailydemocrat.com/content/secret-bias-hidden-mortgage-approval-algorithms	en	FALSE	finances, housing, ren	175953	decaturdailydemocrat.com	http://decaturdailydemocrat.com/	Theoretical	0	
2026047007	8/25/21 12:03	The secret bias hidden in mortgage-approval algorithms	https://www.dailyunion.com/news/nation_world/the-secret-bias-hidden-in-mortgage-approval-algorithms/article_fc691167-f817-53bc-8176-a9ad	en	FALSE	finances, housing	89063	dailyunion.com	http://dailyunion.com/#spider	Theoretical	0	
2029009690	8/25/21 18:03	The secret bias hidden in mortgage-approval algorithms	https://www.beaumontenterprise.com/news/article/The-secret-bias-hidden-in-mortgage-approval-16410286.php	en	FALSE	finances, housing	663403	Jasper Newsboy	http://www.beaumontenterprise.com/jasper/	Theoretical	0	
2025499728	8/25/21 6:56	The secret bias hidden in mortgage-approval algorithms	https://www.seattlepi.com/news/article/The-secret-bias-hidden-in-mortgage-approval-16410286.php	en	FALSE	finances, housing	32770	blog.seattlepi.com	http://blog.seattlepi.com#spider	Theoretical	0	
2025498642	8/25/21 7:13	The secret bias hidden in mortgage-approval algorithms	https://abcnews.go.com/Business/wireStory/secret-bias-hidden-mortgage-approval-algorithms-79633917	en	FALSE	finances, housing	39000	ABC News	http://abcnews.go.com/	Theoretical	0	
2025597073	8/25/21 11:24	The secret bias hidden in mortgage-approval algorithms	https://nypost.com/2021/08/25/the-secret-bias-hidden-in-mortgage-approval-algorithms/	en	FALSE	finances, housing	7	New York Post	http://www.nypost.com/	Theoretical	0	

1298379251	4/6/19 0:00	The Secret Trust Scores Companies Use to Judge Us All	https://www.wsj.com/articles/the-secret-trust-scores-companies-use-to-judge-us-all-11554523206	en	FALSE		1150	Wall Street Journal	https://www.wsj.com/	Theoretical	0	
1205712854	2/23/19 10:00	The Willow breast pump eases some of the worst parts of pumping, for a price	http://feedproxy.google.com/~r/Mashable/~3/gid2nj4EhIc/	en	FALSE		5527	Mashable!	http://mashable.com	Theoretical	0	
2026971591	8/27/21 5:00	There Is Secret Bias Hidden in Mortgage-Approval Algorithms	https://www.omahadailyrecord.com/content/there-secret-bias-hidden-mortgage-approval-algorithms	en	TRUE	finances, housing	661934	Omaha Daily Record	http://www.omahadailyrecord.com/	Theoretical	0	
1022274954	10/12/18 4:28	There's a Good Chance Your Relatives' DNA Is Online. That Means People Can Find You, To	https://www.livescience.com/63818-easy-identify-people-genetic-databases.html	en	FALSE		20410	livescience.com	http://www.livescience.com	Theoretical	0	
1364186691	8/13/19 13:53	These dating apps were found to be leaking users’ exact locations	https://www.dailydot.com/debug/dating-apps-leaking-users-exact-locations/	en	FALSE		366828	Daily Dot	http://dailydot.com/	Case	0	
1844733400	2/6/21 14:34	They Stormed the Capitol. Their Apps Tracked Them	https://mobile.slashdot.org/story/21/02/06/0256208/they-stormed-the-capitol-their-apps-tracked-them?utm_source=rss1.0mainlinkanon&utm_m	en	FALSE		663527	San Antonio News	http://sanantonionews.com/	Case	0	
1830589306	1/21/21 22:38	This Start-Up Founder Sees A Data Privacy Reckoning On The Horizon	https://www.forbes.com/sites/hessiejones/2021/01/21/this-start-up-founder-sees-a-data-privacy-reckoning-on-the-horizon/	en	FALSE		1104	Forbes	http://www.forbes.com/	Theoretical	0	
1996938317	7/21/21 12:47	Top Catholic priest resigns after phone data tracked to Grindr	https://nypost.com/2021/07/21/top-catholic-priest-resigns-after-phone-data-tracked-to-grindr/	en	FALSE	computers and the int	7	New York Post	http://www.nypost.com/	Case	1	
1439120520	11/7/19 9:01	TrialAssure Launches First-in-Class Document Anonymization Tool Using Machine Learning a	http://svdaily.com/trialassure-launches-first-in-class-document-anonymization-tool-using-machine-learning-and-artificial-intelligence-to-protect-p	en	FALSE		659489	Silicon Valley Daily	http://www.svdaily.com/	Theoretical	0	
1512718472	2/4/20 2:01	Twitter says a certain someone tried to discover the phone numbers used by potentially millio	https://go.theregister.co.uk/feed/www.theregister.co.uk/2020/02/04/twitter_phone_numbers/	en	FALSE		5532	The Register	http://www.theregister.co.uk/	Case	0	Twitter pho
1998905002	7/23/21 9:07	UVM researchers create trend-prediction software	https://www.wcax.com/2021/07/23/uvm-group-creates-twitter-prototype/	en	FALSE	computers and the int	69923	wcax.com	http://www.wcax.com/#spider	Theoretical	0	
1587047881	4/23/20 12:48	UW releases coronavirus contact-tracing app	http://rssfeeds.krem.com/~/622245112/0/krem/news~UW-releases-coronavirus-contacttracing-app	en	FALSE		89905	krem.com	http://www.krem.com/#spider	Theoretical	0	
1586803819	4/23/20 9:14	UW, Microsoft release coronavirus contact-tracing app	http://rssfeeds.king5.com/~/622233424/0/king5/local~UW-Microsoft-release-coronavirus-contacttracing-app	en	FALSE		19647	king5.com	http://www.king5.com	Theoretical	0	
1543494240	3/9/20 21:21	Vanderbilt student says he tested positive for coronavirus	https://www.wsmv.com/news/vanderbilt-student-says-he-tested-positive-for-coronavirus/article_805abc70-626d-11ea-8407-6f7cff6facdc.html	en	FALSE		71152	wsmv.com	http://www.wsmv.com/#spider	Theoretical	0	
1578452564	4/15/20 2:44	Virus 'tracing' by smartphone: A key to reopening society?	https://www.rawstory.com/2020/04/virus-tracing-by-smartphone-a-key-to-reopening-society/	en	FALSE		1127	Raw Story	http://rawstory.com	Theoretical	0	
577488957	2/7/17 2:30	Vizio To Pay Millions After Secretly Spying On Customers, Selling Viewer Data	http://lbccvikingnews.com/tech/vizio-to-pay-millions-after-secretly-spying-on-customers-selling-viewer-data/81073	en	FALSE		28102	Viking	http://lbccviking.com/	Case	0	Vizio data
2017632581	8/12/21 18:00	What exactly are Bitcoin Mixers?	https://www.eyeonannapolis.net/2021/08/what-exactly-are-bitcoin-mixers/	en	FALSE	privacy, computers an	661144	Eye on Annapolis	http://www.eyeonannapolis.net/	Theoretical	0	
1582076372	4/18/20 11:01	What is contact tracing?	https://techcrunch.com/2020/04/18/what-is-contract-tracing/	en	FALSE		4438	TechCrunch	http://techcrunch.com	Theoretical	0	
1582243434	4/18/20 11:01	What is contact tracing?	https://api.follow.it/track-rss-story-click/Q3dp_Nkg6aApXQL5mzAkxKn2y1ES5B5v19-cryXjmpJ8-NBGn1kZvt_98q38_AK3iSbMgUv_gOyKQ-JFYI	en	FALSE		31448	monroviaweekly.com	http://www.monroviaweekly.com#spider	Theoretical	0	
1582269713	4/18/20 11:01	What is contact tracing?	https://api.follow.it/track-rss-story-click/Q3dp_Nkg6aApXQL5mzAkxKn2y1ES5B5v19-cryXjmpJ8-NBGn1kZvt_98q38_AK3iSbMgUv_gOyKQ-JFYI	en	FALSE		34587	pasadenaindependent.com	http://www.pasadenaindependent.com#spider	Theoretical	0	
1582269361	4/18/20 11:01	What is contact tracing?	https://api.follow.it/track-rss-story-click/Q3dp_Nkg6aApXQL5mzAkxKn2y1ES5B5v19-cryXjmpJ8-NBGn1kZvt_98q38_AK3iSbMgUv_gOyKQ-JFYI	en	FALSE		131661	morgannews.com	http://morgannews.com/	Theoretical	0	
1368742286	8/19/19 12:32	What New Methods Are Being Used To Protect User Data?	https://www.forbes.com/sites/quora/2019/08/19/what-new-methods-are-being-used-to-protect-user-data/	en	FALSE		1104	Forbes	http://www.forbes.com/	Research	0	
1289580606	5/21/19 20:39	What we know so far about Trump's tax returns, explained	https://www.vox.com/2019/5/21/18634939/trump-tax-returns-irs-memo	en	FALSE		104828	Vox	http://www.vox.com	Case	1	Trump tax
1998845248	7/23/21 8:59	When Catholic media meets tabloid aggressiveness, we all lose	https://angelusnews.com/news/nation/when-catholic-media-meets-tabloid-aggressiveness-we-all-lose/	en	FALSE	christians and christiar	194140	angelusnews.com	http://angelusnews.com/	Case	1	Catholic p
1576148269	4/12/20 7:26	Will We Accept Being Monitored Via Our Smartphones As A Way Out Of The Pandemic, And	https://www.forbes.com/sites/enriquedans/2020/04/12/will-we-accept-being-monitored-via-our-smartphones-as-a-way-out-of-the-pandemic-and	en	FALSE		1104	Forbes	http://www.forbes.com/	Theoretical	0	
1597371319	5/5/20 9:00	Withings Lockdown Lowdown Study Shows People Are Not Gaining Weight Or Slowing Dow	https://www.thestreet.com/press-releases/withings-lockdown-lowdown-study-shows-people-are-not-gaining-weight-or-slowing-down-during-sel	en	FALSE		21619	The Street	http://www.thestreet.com	Theoretical	0	
583140439	2/16/17 10:27	Would you share your personal tax data for a deal on a loan?	http://www.dailyherald.com/article/20170219/business/170219154/	en	FALSE		104	Daily Herald - Arlington Heights, IL	http://www.dailyherald.com/	Theoretical	0	
794062810	1/20/18 9:00	WTF is GDPR?	https://techcrunch.com/2018/01/20/wtf-is-gdpr/	en	FALSE		4438	TechCrunch	http://techcrunch.com	Case	0	
981753253	9/10/18 16:51	You Discovered Your Genetic History. Is It Worth the Privacy Risk?	http://fortune.com/2018/09/10/genetic-history-test-privacy-risk/	en	FALSE		1105	Fortune	http://fortune.com	Theoretical	0	
1543022179	3/9/20 10:00	Your Fitbit Could Be Used to Monitor the Spread of Coronavirus. Is That Really What We War	https://pjmedia.com/trending/your-fitbit-could-be-used-to-monitor-the-spread-of-coronavirus-is-that-really-what-we-want/	en	FALSE		19082	PJ Media	http://pjmedia.com	Theoretical	0	
1053424344	2/12/17 10:19	Your Vizio TV was probably spying on you, says FTC	http://www.kcra.com/article/your-vizio-tv-was-probably-spying-on-you-says-ftc/8734022	en	FALSE		29811	kcra.com	http://www.kcra.com#spider	Case	0	
1056897428	2/12/17 10:19	Your Vizio TV was probably spying on you, says FTC	http://www.kcci.com/article/your-vizio-tv-was-probably-spying-on-you-says-ftc/8734022	en	FALSE		21605	kcci.com	http://www.kcci.com	Case	0	
1049847304	2/12/17 10:19	Your Vizio TV was probably spying on you, says FTC	http://www.wlwt.com/article/your-vizio-tv-was-probably-spying-on-you-says-ftc/8734022	en	FALSE		78560	wlwt.com	http://www.wlwt.com/#spider	Case	0	
