# Supplementary material for: Global healthcare fairness: We should be sharing more, not less, data
Source: PLOS Digit Health. 2022 Oct 6;1(10):e0000102. doi: 10.1371/journal.pdig.0000102 (PMC9931202; doi:10.1371/journal.pdig.0000102)
Supplement: S3 Table — (PDF) [file pdig.0000102.s003.pdf]

Complete List of individual cases on media about personal information disclosure

5 years, 09-01-2016 through 09-01-2021

**Characteristic**	**N = 23**[1]
title	
A Catholic priest resigned after a news site used cell phone location data to track his phone	1 (4.3%)
Catholic priest quits after “anonymized” data revealed alleged use of Grindr	1 (4.3%)
Feds Torpedo Massive Dark Web Child Porn Site	1 (4.3%)
Fugitive Cop Says He’s Behind the DNC Leaks. It’s His Latest Hoax.	1 (4.3%)
Inside the Industry That Unmasks People At Scale	1 (4.3%)
IRS Followed Bitcoin Transactions, Resulting In Takedown Of The Largest Child Exploitation Site On The Web	1 (4.3%)
Msgr. Burrill’s Resignation and the Surveillance Age: 5 Points to Consider	1 (4.3%)
New Catholic website 'The Pillar' operates on shaky journalistic foundation	1 (4.3%)
Opinion | Where Even the Children Are Being Tracked - The New York Times	1 (4.3%)
Privacy breaches in University file system affect 200 people	1 (4.3%)
Private Browsing Really Anonymous? Incognito Mode Histories Exposed By Researchers' Fake Company	1 (4.3%)
The battle over Trump’s tax returns, explained	1 (4.3%)
These dating apps were found to be leaking users’ exact locations	1 (4.3%)
They Stormed the Capitol. Their Apps Tracked Them	1 (4.3%)
Top Catholic priest resigns after phone data tracked to Grindr	1 (4.3%)
Twitter says a certain someone tried to discover the phone numbers used by potentially millions of twits	1 (4.3%)
Vizio To Pay Millions After Secretly Spying On Customers, Selling Viewer Data	1 (4.3%)
What we know so far about Trump’s tax returns, explained	1 (4.3%)
When Catholic media meets tabloid aggressiveness, we all lose	1 (4.3%)
WTF is GDPR?	1 (4.3%)
Your Vizio TV was probably spying on you, says FTC	3 (13%)

[1] n (%)
